# Biomimetic nanoflowers by self-assembly of nanozymes to induce intracellular oxidative damage against hypoxic tumors

**DOI:** 10.1038/s41467-018-05798-x

**Published:** 2018-08-20

**Authors:** Zhenzhen Wang, Yan Zhang, Enguo Ju, Zhen Liu, Fangfang Cao, Zhaowei Chen, Jinsong Ren, Xiaogang Qu

**Affiliations:** 1 Laboratory of Chemical Biology and State Key Laboratory of Rare Earth Resources Utilization, Changchun Institute of Applied Chemistry, Chinese Academy of Sciences, Changchun, Jilin 130022 China; 2 University of Chinese Academy of Sciences, Beijing, 100039 China

## Abstract

Reactive oxygen species (ROS)-induced apoptosis is a promising treatment strategy for malignant neoplasms. However, current systems are highly dependent on oxygen status and/or external stimuli to generate ROS, which greatly limit their therapeutic efficacy particularly in hypoxic tumors. Herein, we develop a biomimetic nanoflower based on self-assembly of nanozymes that can catalyze a cascade of intracellular biochemical reactions to produce ROS in both normoxic and hypoxic conditions without any external stimuli. In our formulation, PtCo nanoparticles are firstly synthesized and used to direct the growth of MnO_2_. By adjusting the ratio of reactants, highly-ordered MnO_2_@PtCo nanoflowers with excellent catalytic efficiency are obtained, where PtCo behaves as oxidase mimic and MnO_2_ functions as catalase mimic. In this way, the well-defined MnO_2_@PtCo nanoflowers not only can relieve hypoxic condition but also induce cell apoptosis significantly through ROS-mediated mechanism, thereby resulting in remarkable and specific inhibition of tumor growth.

## Abstract (web summary)

Hypoxic tumors are resistant to dynamic therapy, limiting potential treatment options. Here, the authors describe a nanoflower where oxidase mimicking PtCo nanoparticles are decorated with catalase mimicking MnO_2_ to reverse tumor hypoxia and generate reactive oxygen species for dynamic therapy.

## Introduction

Reactive oxygen species (ROS) are highly reactive ions and free radicals, including singlet oxygen (^1^O_2_), superoxide radicals (O_2_^•−^), hydroxyl radicals (•OH), and peroxides (O_2_^2−^), which play imperative roles in numerous physiological processes^1,2^. At lower concentrations, ROS can function as a crucial second messenger to modulate cell signaling, adhesion, migration, and homeostasis^3^. Whereas elevated ROS levels have the capacity to oxidize unsaturated fatty acids in lipids and amino acids of proteins, inducing irreversible damage to vital organelles and DNA, ultimately leading to cell apoptosis and necrosis^4^. Of particular note, owning to different redox states between cancer and normal cells, malignant cells work with a heighted basal level of ROS-mediated signaling, making them more susceptible to the detrimental effects of exogenous ROS than normal cells^5,6^. Therefore, extensive research efforts have been directed towards the design of ROS-generating systems to induce intracellular oxidative stress for cancer therapy.

Particularly, photodynamic therapy (PDT), which employs light to activate photosensitizers to generate ROS, has been considered as the most promising treatment option because of its minimal invasiveness, site-specific activation, and negligible drug resistance^7–9^. However, the relatively low tissue-penetrating depth of light in biological tissue still remains as an intrinsic shortcoming^10–12^. More troublesome, the reliance of PDT on molecular oxygen limits its effectiveness in hypoxic environments, including many solid tumors^13–15^. Alternatively, sonodynamic therapy (SDT) that comprises ultrasound and sonosensitzer as ROS generator has been developed recently and witnessed some exciting results^16,17^. Although promising, such strategy would induce hyperthermia effect to surrounding normal tissues as well as confer the treatment outcomes with respectable complexity and variability. Therefore, it is highly desired to develop a paradigm that not only can directly generate exogenous ROS inside tumors without any external stimulus, but also overcome the hypoxic microenvironment of tumors for magnifying ROS generation.

Nanozymes that integrate the functions of both nature enzymes and nanomaterials have sparked increasing interest due to their higher stability against stringent conditions, controlled synthesis at low cost and tunable catalytic activities^18–20^. Such unique advantages make nanozymes widely exploit in biomedical applications, such as biosensing, disease diagnosis, tissue engineering, etc.^21–23^. Recently, a special focus has been made on probing the ability of nanozymes in controlling intracellular biochemical reactions to treat various diseases including anti-aging, stroke, and inflammation^24,25^. For example, V_2_O_5_, Prussian blue and Fe_3_O_4_ nanomaterials with antioxidant enzyme-like activity under neutral pH condition have been demonstrated to catalyze the reduction of cytotoxic ROS into non-toxic products inside cells and prevent them against oxidative stress^26–29^. However, along this line, the strong oxidizing capacity of nanozymes in acidic conditions has long been ignored, particularly in the field of cancer therapy. Concerning the acidic tumor microenvironment and the nanozyme’s ability to modulate intracellular biochemical processes, we envision that the nanozymes-based platform can realize tumor-specific therapy and resolve the lingering problems of current ROS-mediated therapy systems.

**Fig. 1:**
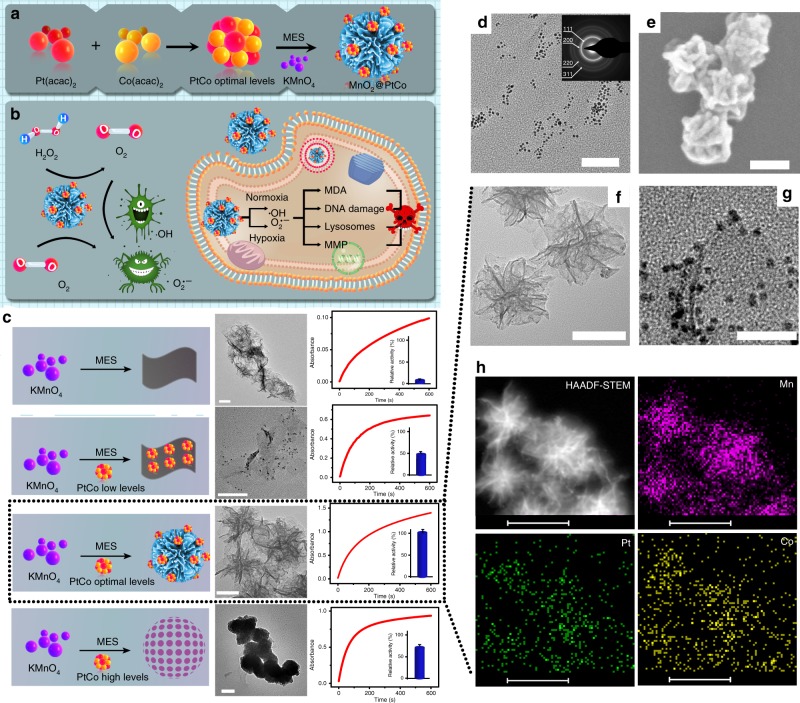
Synthesis of MnO_2_@PtCo nanoflowers. **a** Schematic illustration showing the self-assembly of nanozymes into well-defined nanoflowers. **b** Schematic representation of the generation mechanism of ROS and cytotoxicity by MnO_2_@PtCo nanoflowers under different oxygen tensions. **c** The influence of PtCo concentration on the morphology and catalytic performance of MnO_2_@PtCo assemblies is directly suggested by TEM images and UV–vis analysis. In the lower concentration, PtCo nanoparticles could promote and stabilize MnO_2_ crystal to form two-dimensional nanosheets. With increasing PtCo concentration, it could induce the self-assembly of MnO_2_@PtCo nanosheets to form nanoflower. Nevertheless, further increasing the concentration of PtCo would lead to an increase in the level of self-assembly and therefore cause the formation of aggregated MnO_2_@PtCo nanospheres. Scale bars are 50 nm. Data were presented as mean ± s.d. (*n* = 5). **d** TEM image of PtCo nanozymes (inset: SAED pattern). Scale bar is 50 nm. SEM (**e**) and TEM (**f**) image of MnO_2_@PtCo nanoflowers. Scale bars are 100 nm. **g** HRTEM image of MnO_2_@PtCo nanoflowers. Scale bar is 20 nm. **h** The high-angle annular dark-field (HAADF)-STEM image of MnO_2_@PtCo nanoflowers, and corresponding TEM element mappings of the Mn K-edge, K-edge Pt, and Co K-edge signals. Scale bars are 100 nm

Herein, we construct a biomimetic nanoflower based on self-assembly of nanozymes that is capable of generating ROS in both normoxic and hypoxic conditions with a noninvasive and facile manner, where any external stimulus is not needed. In our design, PtCo nanoparticles behave as oxidase mimics whereas MnO_2_ is used to mimic catalase (CAT), both of which assemble into high-ordered nanoflowers by simply adjusting the amount of reactants (Fig. 1a). PtCo nanozyme can catalyze the oxidation reaction cascades to induce intracellular oxidative damage, thereby leading to efficient therapeutic outcome. Meanwhile, with the intrinsic CAT-like activity, MnO_2_ component has the ability to induce the decomposition of H_2_O_2_ into O_2_ rapidly and efficiently, which not only surmounts the intrinsic hypoxic environment of tumors but, importantly, makes our therapeutic system independent on O_2_. In this way, chemodynamic therapy is readily realized by utilizing endogenous chemical energy to generate ROS, without the need for external energy input and local oxygen, thus circumventing the limitations posed by the PDT and SDT (Fig. 1b). Both in vitro and in vivo experiments demonstrate that MnO_2_@PtCo nanoflowers dramatically induce intracellular oxidative damage under different oxygen tensions as well as inhibit tumor growth in xenograft-bearing mice. More importantly, the pH-dependent catalytic functions of nanozymes make the therapeutic outcome specificity for tumors, leaving normal tissues unharmed. Therefore, we believe that the present study may open up a leverage for cancer therapy and ignite the further explorations and applications of nanozymes in various fields, such as bioimaging, detoxification and catalysis.

## Results

### Design and construction of MnO_2_@PtCo nanoflowers

To substantiate our design, PtCo nanozyme was specially chosen as oxidase mimic on account of its advantages of easy preparation, excellent catalytic ability in a broad pH range, smaller size, good dispersity and stability compared with other oxidase mimics^19,30–32^. We initially studied the influence of different atomic ratio Pt/Co on the resultant nanoparticles’ enzyme-mimic activity. As illustrated in Supplementary Fig. 1a, b, when the atomic ratio of Pt/Co was 3:1, PtCo nanoparticles exhibited the best catalytic activity, which was consistent with previous report^32^. Moreover, no significant Co^2+^ ions were released in cell culture medium and blood sample after incubation of 8, 24, and 48 h (Supplementary Fig. 1c), indicating a good stability. Consequently, PtCo nanoparticles at the molar ratio of 3:1 were used in our work. A typical transmission electron microscopy (TEM) image showed that these well-prepared PtCo nanoparticles were uniform in size with a diameter of around 3 nm (Fig. 1d). The interfringe distance of lattice fringes in high-resolution TEM (HRTEM) image and the characteristic diffraction spots in selected area electron diffraction (SAED) pattern vividly revealed the formation of the bimetallic phase of Pt and Co, which further confirmed by X-ray diffraction (XRD) peak and X-ray photoelectron spectroscopy (XPS) analysis (Supplementary Fig. 2). To obtain MnO_2_@PtCo assemblies, the PtCo nanoparticles were utilized to direct the growth of MnO_2_. Intriguingly, we found that the amount of PtCo played a crucial role in the formation of MnO_2_@PtCo. As illustrated in Fig. 1c, without PtCo or higher concentrations of PtCo presented, the resulting MnO_2_@PtCo nanomaterials tended to aggregate and form precipitates, both of which exhibited low catalytic capacity. Despite the well-dispersed MnO_2_@PtCo nanosheets were obtained in the presence of low dose of PtCo, the lower enzymatic activity was observed. Comparatively, when increasing the dose of PtCo to optimal level, the assembly of nanozymes in a hierarchical manner with intricate flower-like nanoarchitecture was realized (Fig. 1c, e, f), which provided a significant impetus in mimicking the structure of nature enzyme^33^, thereby offering the highest catalytic performance. Moreover, the enlarged picture of MnO_2_@PtCo nanoflowers in Fig. 1g demonstrated that PtCo nanoparticles were uniformly dispersed on the surface of nanoflower. The mechanism of PtCo to drive the self-assembly of MnO_2_@PtCo nanomaterials may arise from PtCo nanoparticles’ abundant surface functional groups (Supplementary Fig. 3), which endows them with the ability to behave as the heterogeneous nucleation sites for MnO_2_ growth^34^. To investigate the state of Mn in the nanoflowers, XPS analysis was performed. Binding-energy peaks were observed for Mn 2*p*_1/2_ and Mn 2*p*_3/2_ at 641.9 and 653.6 eV respectively (Supplementary Fig. 4), which were typical values for Mn^4+^, suggesting the formation of MnO_2_^35^. Meanwhile, uniform distribution of Mn, Pt, and Co in Fig. 1h further illustrated the successful formation of MnO_2_@PtCo multi-nanozymes. The content ratio of MnO_2_ to PtCo in MnO_2_@PtCo nanoflowers was determined to be 4:1 by energy dispersive X-ray spectroscopy (EDX) analysis (Supplementary Fig. 5). Besides, dynamic light scattering (DLS) data showed that the resultant nanoflowers had a good dispersity with an average diameter of about 200 nm, which did not change during 7 days of incubation in both PBS and DMEM containing 10% fetal bovine serum (FBS), as illustrated in Supplementary Fig. 6. Moreover, the morphology of MnO_2_@PtCo nanoflowers has little change after 7 days’ incubation (Supplementary Fig. 7), illustrating their long-term stability in culture media.

### Catalytic ability of MnO_2_@PtCo nanoflowers

**Fig. 2:**
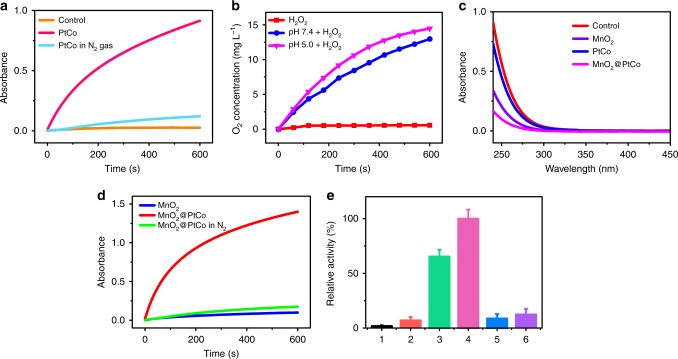
Multi-enzyme activity of MnO_2_@PtCo nanoflowers in normoxia. **a** The study of PtCo nanozymes’ oxidase-like activity. Time-dependent absorbance changes at 652 nm of TMB in the presence of PtCo at N_2_ gas (mimicking hypoxic condition) or not. **b** Oxygen generation in H_2_O_2_ solution with MnO_2_ added under different pH values (7.4 and 5.0). **c** The study of MnO_2_ component’s CAT-like activity. UV–vis spectra of remain H_2_O_2_ were recorded after reacting with different nanomaterials. **d** The study of MnO_2_@PtCo nanoflowers’ oxidase-like activity. Time-dependent absorbance changes at 652 nm of TMB with different treatments. **e** The qualitative analysis of different nanomaterials’ oxidase activity. 1: control; 2: MnO_2_; 3: PtCo; 4: MnO_2_@PtCo; 5: PtCo in N_2_ gas; 6: MnO_2_@PtCo in N_2_ gas. Data were presented as mean ± s.d. (*n* = 5)

The oxidase-like activity was firstly investigated by real-time monitoring the change in the substrate 3,3,5,5′-tetramethylbenzidine (TMB) absorption at 652 nm. As illustrated in Fig. 2a, PtCo nanoparticles could effectively catalyze TMB oxidation in normoxia, whereas in hypoxia, their catalytic ability was vanished, revealing PtCo component has oxidase-like activity. Note that most tumors tend to develop a hypoxic region^36^, where catalytic capacity of PtCo nanozyme will be compromised. To surmount this problem, MnO_2_ nanomaterials with intrinsic CAT-like activity were employed^29^. Moreover, MnO_2_ could also react with H_2_O_2_ and H^+^ to produce O_2_^37^, thereby holding great attractive to overcome tumor hypoxia. As shown in Fig. 2b and Supplementary Fig. 8, clear gas bubbles and dissolved O_2_ were observed after H_2_O_2_ (100 μM, mimicking tumor microenvironment) was added into tubes containing MnO_2_ under both pHs (7.4, 5.0). Moreover, the concentration of H_2_O_2_ was decreased in the sample containing MnO_2_ components significantly (Fig. 2c), indicating MnO_2_ could function as a good CAT mimic. In parallel to mimicking CAT, MnO_2_ components also possessed peroxidase-like activity in a pH-dependent manner (Supplementary Fig. 9a, b), in accordance with previous reports^38,39^. Subsequently, we embarked on investigating which kind of enzyme activity MnO_2_ showed in a tumor-mimicking microenvironment (pH 6.8, H_2_O_2_ 100 μM). As presented in Supplementary Fig. 9c, d, the concentration of oxidized TMB was calculated to be 16.7 μM by the Beer–Lambert Law, indicating 16.7% of H_2_O_2_ was involved in the peroxidase reaction of MnO_2_. Comparatively, 70.6% of H_2_O_2_ was involved in the CAT reaction of MnO_2_, suggesting MnO_2_ mainly exhibited CAT-like activity in tumor microenvironment. Benefiting from CAT-like activity of MnO_2_ and oxidase-like activity of PtCo, MnO_2_@PtCo nanoflowers exerted both significant CAT (Fig. 2c) and oxidase-like activity (Fig. 2d), and higher than their counterparts (Fig. 2e). The corresponding control experiments demonstrated that the higher enzymatic activity of our nanoflowers was not stemmed from simply physical mixing (Supplementary Fig. 10a, b), which may be attributed to the larger surface area and confinement effect of nanoflowers. Importantly, the catalytic capacity of MnO_2_@PtCo nanoflowers not only enhanced with the increasing concentration but also possessed broad substrate recognition ability (Supplementary Fig. 10c, d).

**Fig. 3:**
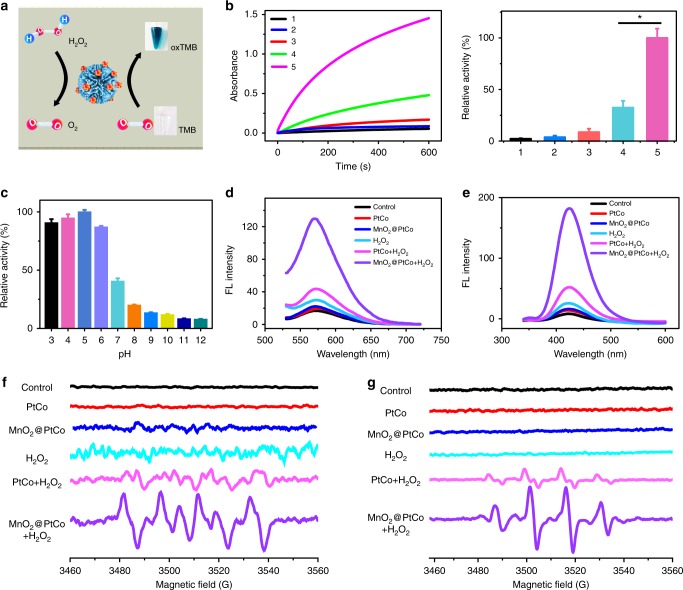
Multi-enzyme activity and catalytic mechanism of MnO_2_@PtCo nanoflowers in hypoxia. **a** Schematic illustration of catalytic reactions of MnO_2_@PtCo nanoflowers in the hypoxic H_2_O_2_ condition. **b** The oxidase-like activity of various nanomaterials by monitoring the absorption of TMB at 652 nm and the qualitative analysis of their catalytic activity in the hypoxic H_2_O_2_ (100 μM) environment. 1: H_2_O_2_ alone; 2: PtCo; 3: MnO_2_@PtCo; 4: PtCo + H_2_O_2_; 5: MnO_2_@PtCo + H_2_O_2_. Asterisks indicate significantly differences (^*^P < 0.01, ^**^P < 0.005, ^***^P *<* 0.001), analyzed by unpaired Student’s two-sided *t* test. **c** The influence of pH on the catalytic ability of MnO_2_@PtCo nanoflowers. **d** Fluorescence spectra of hydroethidine incubated with MnO_2_@PtCo nanoflowers in the hypoxic H_2_O_2_ (100 μM) condition to demonstrate the presence of O_2_^•−^. **e** Fluorescence spectra of TA incubated with MnO_2_@PtCo nanoflowers in the hypoxic H_2_O_2_ (100 μM) condition to demonstrate the generation of •OH. **f** ESR spectra of BMPO/•OOH adducts from different groups in the hypoxic H_2_O_2_ (100 μM) condition upon addition of DMSO. **g** ESR spectra of BMPO/•OH adducts were collected from different samples in the hypoxic H_2_O_2_ (100 μM) condition upon addition of SOD. Data were presented as mean ± s.d. (*n* = 5)

Inspired by the CAT-like and oxidase-like activity of MnO_2_@PtCo, we then investigated whether these multi-nanozyme assemblies could catalyze the cascade oxidation reactions in hypoxia. Since MnO_2_@PtCo nanoflowers possessed CAT-like and peroxidase-like activity in an acidic H_2_O_2_ condition, we initially evaluated which kind of enzyme activity MnO_2_@PtCo nanoflowers showed. As shown in Supplementary Fig. 11, MnO_2_@PtCo nanoflowers primarily exhibited CAT-like activity in tumor microenvironment, which could couple with the oxidative reaction of nanoflowers to generate ROS and then induce cellular oxidative damage. As expected, MnO_2_@PtCo nanoflowers exhibited excellent oxidative ability in the hypoxic H_2_O_2_ environment (Fig. 3b), which could be attributed to the cooperation of between CAT and oxidase of nanoflowers. Moreover, the quantitative analysis showed that the relatively catalytic activity of MnO_2_@PtCo nanoflowers was three times higher than that of PtCo nanozymes, demonstrating our nanoflowers were O_2_ self-supplying catalysts by taking tumor microenvironment. Furthermore, the effect of pH and temperature on the catalyze activity of MnO_2_@PtCo nanoflowers as well as their catalytic stability under physiological conditions were investigated (Fig. 3c and Supplementary Figs. 12, 13). We found that our nanoflowers not only exhibited high activity over a broad pH (2.5–6.8) and temperature range but also retained significant enzymatic activity in an intracellular microenvironment, providing an essential prerequisite for subsequent cancer therapy. More importantly, MnO_2_@PtCo nanoflowers still possessed outstanding catalytic ability over 7 days of incubation in both PBS buffer (~92%) and cell culture medium (~87%), as demonstrated in Supplementary Fig. 14.

### Catalytic mechanism of MnO_2_@PtCo nanoflowers

To elucidate the catalytic mechanism of MnO_2_@PtCo nanoflowers, the possible produced active intermediates during the reaction were determined. A dramatic increase in fluorescence intensity of hydroethidine, one highly selective fluorescence probe for O_2_^•−^^10^, was observed for MnO_2_@PtCo nanoflowers treatment (Supplementary Fig. 15), demonstrating that their capability to generate O_2_^•−^. Besides, significant fluorescence enhancement of 2-hydroxyterephtthalic acid (HA) was also seen (Supplementary Fig. 16), suggesting •OH production. Conversely, a few amount of ^1^O_2_ was generated when MnO_2_@PtCo nanoflowers incubated with 1,3-diphenylisobenzofuran (DPBF) indicator, even increasing their concentrations to 100 μg mL^−1^ (Supplementary Fig. 17). To identify which kind of ROS played a critical role in our system, one broad dichlorofluorescein diacetate (DCFH-DA) ROS probe and specific ROS scavengers DMSO (•OH), superoxide dismutase (SOD, O_2_^•−^), and NaN_3_ (^1^O_2_) were used. As displayed in Supplementary Fig. 18, no obvious fluorescence change was observed upon adding NaN_3_ (11%), whereas the fluorescence intensity of DCFH-DA was diminished dramatically after treatment with DMSO (46%) or SOD (40%), indicating that the oxidative ability of MnO_2_@PtCo nanoflowers primarily stemmed from •OH and O_2_^•−^. Furthermore, only ~5% of fluorescence intensity remained after subtracting the fluorescence intensity of DCFH-DA generated by •OH, O_2_^•−^, and ^1^O_2_ from the total fluorescence intensity, indicating little amount of other reactive species presented in our system. Furthermore, under the hypoxic H_2_O_2_ condition, a remarkable amount of O_2_^•−^ and •OH were detected by adding MnO_2_@PtCo nanoflowers (Fig. 3d, e). In comparison, no obvious signal of O_2_^•−^ and •OH were detected in MnO_2_ and PtCo presented.

Since electron spin resonance (ESR) spectrum is considered as more direct evidence to identify ROS^40^, 5-tertbutoxycarbonyl-5-methyl-1-pyrroline N-oxide (BMPO) was employed as a trapping probe to further demonstrate the presence of O_2_^•−^ and •OH in hypoxia. Notably, BMPO/•OOH and BMPO/•OH have overlapping ESR spectra^41^, thereby DMSO and SOD scavenger were used during measurement processes. As shown in Fig. 3f, MnO_2_@PtCo nanoflowers induced the generation of a four-line spectrum with relative intensities of 1:1:1:1, which was the characteristic spectrum of BMPO/•OOH. Furthermore, the characteristic spectrum of BMPO/•OH adduct showing a four-line spectrum of 1:2:2:1 intensity was also observed after MnO_2_@PtCo nanoflowers treatment (Fig. 3g). These results validated that our nanoflowers have indeed the potential to generate O_2_^•−^ and •OH and their possibly catalytic mechanisms are provided in Supplementary Fig. 19.

We then investigated the ROS generation ability of MnO_2_@PtCo nanoflowers in both normoxia and hypoxia. Upon increasing the levels of nanoflowers, the amount of generated ROS increased remarkably irrespective of O_2_ status (Supplementary Fig. 20a, d). Moreover, the ROS generating ability enhanced significantly when prolonging the reaction time (Supplementary Fig. 20b, c). Taken together, by engineering self-assembly of nanozymes, our well-designed nanoflowers could generate ROS efficiently in both normoxia and hypoxia without any external stimulus, transcending traditional ROS-mediated treatment paradigms.

### In vitro cytotoxicity of MnO_2_@PtCo nanoflowers

**Fig. 4:**
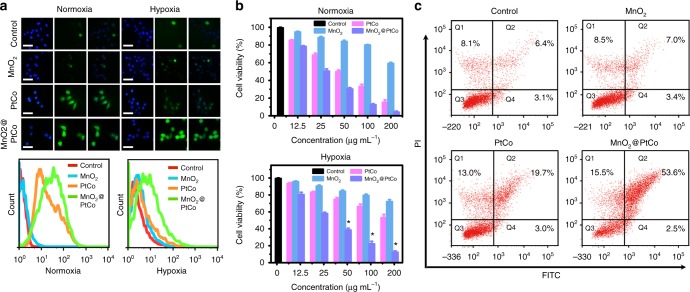
In vitro assessments of MnO_2_@PtCo nanoflowers-induced intracellular oxidative damage. **a** Detection of MnO_2_@PtCo nanoflowers-induced ROS in 4T1 cells in normoxic and hypoxic culture media by fluorescence microscopy and flow cytometry, respectively. The left, middle and right images show DAPI (blue), DCFH-DA (green) and the merged images, respectively. The left, middle and right images show DAPI (blue), DCFH-DA (green) and the merged images, respectively. Scale bars are 50 μm. **b** Cell viability of 4T1 cells treated with different concentrations of nanoparticles for 48 h in different O_2_ tensions. Asterisks indicate significant differences (^*^P < 0.01, ^**^P < 0.005, ^***^P *<* 0.001), analyzed by unpaired Student’s two-sided *t* test. Data were presented as mean ± s.d. (*n* = 5). **c** Fluorescein-annexin V and PI staining assays of 4T1 cells treated with different formulations

Compared with 4T1 cells incubated at 37 °C, the amount of MnO_2_@PtCo nanoflowers inside cells incubated at 4 °C was obviously decreased, implying that these nanoflowers entered cells mainly through energy-dependent endocytosis (Supplementary Fig. 21). Moreover, the co-localization studies showed that the nanoflowers were mainly accumulated in acidic lysosomes, which was beneficial to improving oxidative capacity of nanoflowers (Supplementary Fig. 22). To evaluate intracellular ROS-generated capacity of nanoflowers, flow cytometry and fluorescence microscopy assays were carried out by utilizing DCFH-DA probe. As presented in Fig. 4a, both PtCo and MnO_2_@PtCo treated cells exhibited bright green fluorescence in normoxia, but only obvious fluorescence was observed for the cells incubated with MnO_2_@PtCo under hypoxia, confirming our nanoflowers could overcome the hypoxic environment to generate ROS efficiently.

Having proved the ROS generation ability of MnO_2_@PtCo nanoflowers, their potential cytotoxicity under different O_2_ tensions was evaluated. As illustrated in Fig. 4b, MnO_2_@PtCo nanoflowers treatment offered obvious cancer cell killing in both normoxia and hypoxia because of efficient generation of ROS. Comparatively, PtCo nanozymes exhibited significant cytototoxicy in normoxia, but no obvious cell death was observed in hypoxia. Similar trends were also obtained from the LIVE (green)/DEAD (red) kit (Supplementary Fig. 23). These results indicated that MnO_2_@PtCo nanoflowers could modulate intracellular biochemical reactions to induce cell death, irrespective of O_2_ status. Furthermore, fluorescein-annexin V and propidium iodide (PI) staining assays identified that the cell toxicity of MnO_2_@PtCo nanoflowers in hypoxia were mainly associated with apoptosis (Fig. 4c). Owning to the pH-dependent catalytic functions of nanozymes and different redox states between cancer and normal cells, MnO_2_@PtCo nanoflowers have little cytotoxicity towards normal NIH 3T3 cells in both normaxic and hypoxic environments (Supplementary Fig. 24).

To demonstrate the merits of our nanoflowers in generating ROS, one conventional photosensitizer methylene blue (MB) was utilized to make a preliminary comparison. For ROS generation, MB exhibited a higher ROS generation ability after light irradiation than MnO_2_@PtCo nanoflowers in normoxia, whereas in hypoxic H_2_O_2_ conditions, the nanoflowers showed much higher ROS generation ability than MB (Supplementary Fig. 25a), which could be attributed to the self-supplying O_2_ capacity of nanoflowers by catalyzing H_2_O_2_. Subsequently, we compared their toxicity against 4T1 cells in different O_2_ tensions. Intriguingly, the cell viability induced by MnO_2_@PtCo nanoflowers was comparable to MB treatment in normoxic tension (Supplementary Fig. 25b), which may be attributed to different ROS generation modes, where MB was mainly through a burst generation of ROS under light irradiation, whereas the continuous generation of ROS was demonstrated for MnO_2_@PtCo nanoflowers without any external stimuli. Moreover, with self-supplied O_2_ ability and continuous generation of ROS, MnO_2_@PtCo nanoflowers showed significant toxicity towards 4T1 cells in hypoxic tension while MB not. To further demonstrate the advantages of our nanoflowers, pork tissues with 4 mm thickness were placed on the top of cell incubation plate. We found that the cell toxicity of MB was diminished dramatically upon light irradiation in both normoxia and hypoxia due to the limited light penetration depth, whereas negligible change was observed for nanoflowers treatment (Supplementary Fig. 25c). Taken together, such chemical generation of ROS taken from biological milieu by nanozymes in a noninvasive manner not only exhibited efficient cancer treatment, but also overcame the limitations of PDT.

### Catalytic therapy effect in multicellular tumor spheroids

**Fig. 5:**
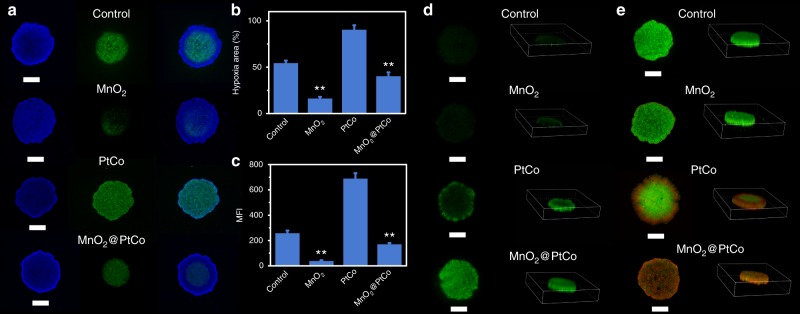
The ability of MnO_2_@PtCo nanoflowers on mitigating hypoxia, generating ROS and cytotoxicity in MCTS. **a** The hypoxia evaluation of 4T1 MCTS at 24 h after various treatments. Hypoxia was assessed by staining with HIF-1α (green) and DAPI (blue). **b** The quantification of hypoxia areas was performed with Image J. **c** Flow cytometric analysis of fluorescence intensity of HIF-1α in MCTS after various treatments. **d** Detection of nanozymes-induced ROS in MCTS. **e** LIVE/DEAD staining of MCTS with Calcein-AM (green) and PI (red) at 24 h after different treatments. Scale bars are 500 μm. Asterisks indicate significant differences (^*^P < 0.01, ^**^P < 0.005, ^***^P *<* 0.001), analyzed by unpaired Student’s two-sided *t* test. Data were presented as mean ± s.d. (*n* = 5)

Since multicellular tumor spheroids (MTCS) could accurately replicate the heterogeneity of tumor microenvironment with a hypoxic core and normoxic surface^42,43^, we then embarked on constructing 4T1 MCTS to investigate the effect of MnO_2_@PtCo nanoflowers on mitigating hypoxia by employing hypoxia inducible factor (HIF)-1α staining assay. There was nearly no green fluorescence was detected in the internal area after 24 h incubation with MnO_2_ nanozyme (Fig. 5a), implying that it could effectively alleviate the hypoxia of MTCS. Comparatively, for PtCo nanozyme, green signals could be clearly seen both in the internal and edge of MCTS due to the continuous O_2_ consumption. Benefiting from reoxygenation capacity of MnO_2_ naozyme, the hypoxia area induced by MnO_2_@PtCo nanoflowers was dramatically decreased to below 40% compared to PtCo nanozyme (Fig. 5b). To quantitatively evaluate the nanoflowers’ ability in lessening hypoxia, flow cytometry analysis was carried out. As displayed in Fig. 5c, MCTS treated with MnO_2_@PtCo nanoflowers exhibited much lower HIF-1α expression than control group, which was in favor for nanoflowers’ oxidative cascade reactions in vivo. With such appealing properties, ROS generation capacity of MnO_2_@PtCo nanoflowers in MTCS was determined. For PtCo treatment, green fluorescence was observed only at MCTS surface, whereas fluorescence signal in MnO_2_@PtCo group was stronger and distributed throughout the entire MCTS (Fig. 5d). Subsequently, we further performed LIVE/DEAD assay of MCTS. After MCTS treated with MnO_2_@PtCo nanoflowers for 24 h, almost all cells in MCTS were destroyed, while the damaged cells were only observed in the edge of MTCS for PtCo nanozyme group due to the inability to overcome the intrinsic hypoxic environment of MTCS (Fig. 5e). These results suggested that our designed MnO_2_@PtCo nanoflowers could initiate intracellular biochemical reactions to alleviate the hypoxic microenvironment of MCTS, generate ROS and ultimately induce their death.

### Cell death mechanism induced by MnO_2_@PtCo nanoflowers

**Fig. 6:**
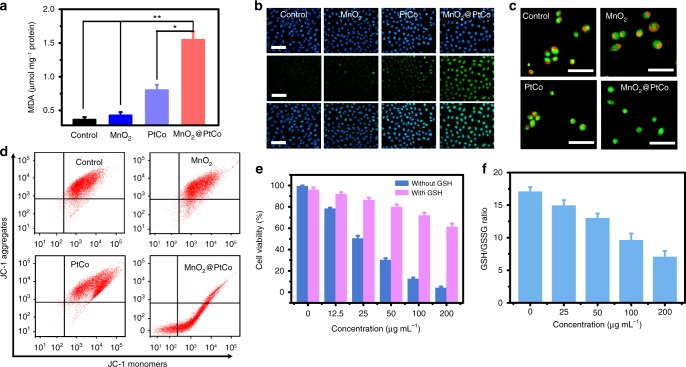
Cell death mechanism caused by MnO_2_@PtCo nanoflowers in hypoxic environment. **a** Changes of MDA content in 4T1 cells following treatment with different formulations. Asterisks indicate significantly differences (^*^P < 0.01, ^**^P < 0.005, ^***^P *<* 0.001), analyzed by unpaired Student’s two-sided *t* test. **b** γ-H_2_AX foci immunofluorescence staining of 4T1 cells incubated with various nanomaterials. The nucleus was stained with DAPI (blue) and γ-H_2_AX (green) was visualized by the FITC-labeled secondary antibody. Scale bars are 100 μm. **c** Fluorescence images of AO staining to evaluate lysosomal integrity of 4T1 cells with different treatments. Scale bars are 200 μm. **d** JC-1 analysis of 4T1 cells as a measure of mitochondrial depolarization after treatment with different formulation. **e** The cell viability incubated with different concentrations of MnO_2_@PtCo nanoflowers with GSH or without GSH added. **f** GSH/GSSH ratios of 4T1 cells treated with different concentrations of MnO_2_@PtCo nanoflowers. Data were presented as mean ± s.d. (*n* = 5)

To investigate whether cell apoptosis caused by MnO_2_@PtCo nanoflowers in hypoxia was through ROS-mediated mechanism, the lipid peroxidation, DNA damage, lysosomes and mitochondrial disruption experiments were conducted. Firstly, thiobarbituric acid (TBA) assay was employed to evaluate lipid peroxidation after different treatments^44^. Compared with untreated control group, the levels of malondialdehyde (MDA) was increased by nearly 400% after exposure to MnO_2_@PtCo nanoflowers, indicative of noticeable lipid peroxidation (Fig. 6a). DNA damage caused by nanoflowers was also evaluated by immunofluorescence staining of γ-H_2_AX, a marker of DNA double-breaks^45^. As shown in Fig. 6b, a remarkable DNA damage was observed for cells treated with MnO_2_@PtCo nanoflowers. Furthermore, acridine orange (AO) was adopted as an indicator for monitoring the integrity of lysosomes^46^. The lysosomes treated with MnO_2_ and PtCo nanozymes emitted bright red fluorescence, while the red fluorescence was remarkably reduced when treated with MnO_2_@PtCo nanoflowers (Fig. 6c), implying the disruption of lysosomal membranes. Subsequently, JC-1 staining was employed to test mitochondrial membrane potential (MMP)^47^. In Fig. 6d, MnO_2_@PtCo nanoflowers led to the increase of red fluorescence aggregates and the decrease of green fluorescence monomer forms inside cells, suggesting the depolarization of mitochondrial membrane. Specially, the ratio of red/green fluorescence intensity caused by MnO_2_@PtCo was approximately three times higher than that of PtCo nanozymes (Supplementary Fig. 26). To further investigate the mechanism of cell death, the ROS scavenger GSH was employed. We discovered that cytotoxicity of MnO_2_@PtCo nanoflowers was vanished upon exogenous addition of GSH (Fig. 6e). More interestingly, MnO_2_@PtCo nanoflowers led to the obvious decrease of intracellular GSH/GSSH ratio, suggesting the imbalance of intracellular redox state (Fig. 6f). All above results demonstrated that MnO_2_@PtCo nanoflowers could trigger cell apoptosis through ROS-mediated mechanism.

### In vivo toxicology of MnO_2_@PtCo nanoflowers

In vivo toxicology of MnO_2_@PtCo nanoflowers was investigated systematically to ensure their safe bioapplications. The liver status was reflected by the levels of alanine transaminase (ALT), aspartate transaminase (AST), alburnin (ALB), globulin (GLB), and total protein (TP), whilst kidney function was evaluated by measuring blood urea nitrogen (BUN) and creatinine (CREA) markers^48^. No significant difference between MnO_2_@PtCo nanoflowers-treated groups and control group was observed at different time points (Supplementary Fig. 27a), elucidating that nanoflowers had little toxicity to liver and kidney. Since the potential risks of MnO_2_@PtCo nanoflowers in inducing inflammatory responses would be reflected in hematological factors, the standard haemotology markers including red blood cells (RBC), hemoglobin (HGB), hematocrit (HCT), mean corpuslar volume (MCV), mean corpuslar hemoglobin (MCH), mean corpuslar hemoglobin concentration (MCHC), white blood cells (WBC,and platelets (PLT) were also measured. Compared with control group, all the blood indexes in the nanoflowers-treated groups were found to be normal, demonstrating no significant infection and inflammation were arose during the whole evaluation period. Besides, the injection of MnO_2_@PtCo nanoflowers had negligible influence toward the mice growth (Supplementary Fig. 27b). Further hematoxylin and eosin (H&E) staining assay of major organs including heart, liver, spleen, lung, and kidney proved the high histocompatibility of MnO_2_@PtCo nanoflowers (Supplementary Fig. 27c). These preliminary in vivo biosafety results indicated MnO_2_@PtCo nanoflowers are nontoxic, which favors their further bioapplications.

### The pharmacokinetics of MnO_2_@PtCo nanoflowers

**Fig. 7:**
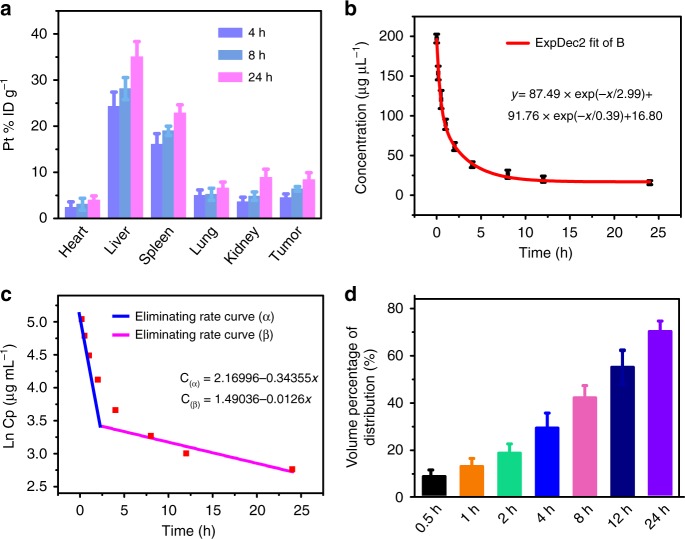
The pharmacokinetics studies of MnO_2_@PtCo nanoflowers. **a** The biodistribution of nanoflowers in main organs and tumors at different time points. **b** The blood circulation curve of intravenously injected nanoflowers. **c** The eliminating rate curve of nanoflowers from the blood circulation curve according to the In(concentration)–*T* relationship. **d** The apparent volume percentage of distribution (*V*_d_%) of intravenously injected nanoflowers. Data were presented as mean ± s.d. (*n* = 3)

The pharmacokinetics of MnO_2_@PtCo nanoflowers was evaluated to elucidate their behaviors in vivo. After systemic intravenous injection (i.v.), the biodistribution analysis of MnO_2_@PtCo nanoflowers at different time points was initially studied. As illustrated in Fig. 7a, the nanoflowers were primarily accumulated in liver and spleen due to the capture of reticuloendothelial system. The relative distribution amounts of nanoflowers within tumor were 4.52% in 4 h and increased against time (6.4%, and 8.42% at time 8 and 24 h, respectively), validating that MnO_2_@PtCo nanoflowers could accumulated at tumor sites by EPR effect. According to blood circulation curve (Fig. 7b), the blood circulation of MnO_2_@PtCo nanoflowers was followed a two-compartment model and their circulating half-life was calculated to be 0.86 h, higher than conventional naked nanoparticles (0.33 h)^49^. Furthermore, based on the In(concentration)–*T* relationship, we calculated that the eliminating curve constants of MnO_2_@PtCo nanoflowers were −0.34355 μg mL^−1^ h^−1^ in the first stage and then decreased to 0.0126 μg mL^−1^ h^−1^ in the second state (Fig. 7c). The apparent volume percentage of distribution of nanoflowers exhibited increasing distribution kinetics in the whole blood of body (Fig. 7d). Collectively, these data indicated that MnO_2_@PtCo nanoflowers exhibited a superior blood circulation profile.

### In vivo tumor therapeutics of MnO_2_@PtCo nanoflowers

**Fig. 8:**
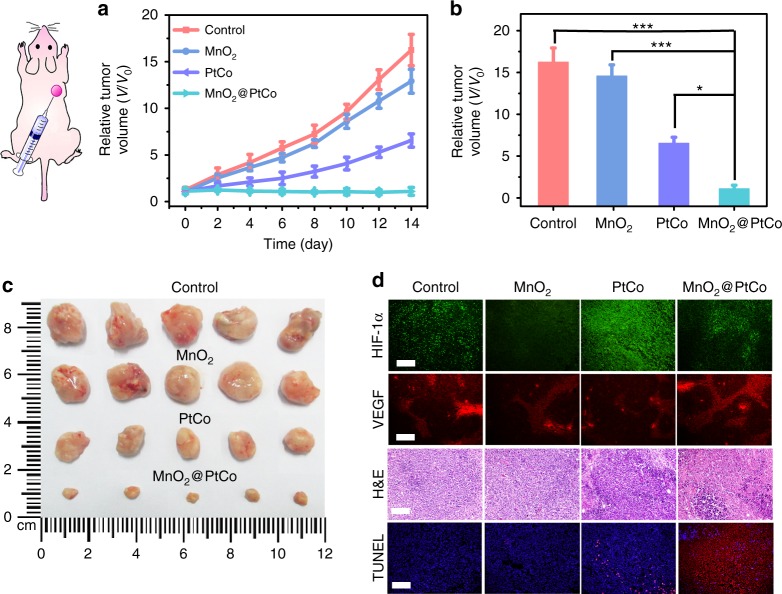
In vivo therapeutic performance of MnO_2_@PtCo nanoflowers by intratumoral injection. **a** Time-dependent tumor volume changes after different treatments. **b** Comparison of inhibition effect of MnO_2_, PtCo, and MnO_2_@PtCo on tumor growth after 14 days post injection. Asterisks indicate significant differences (^*^P < 0.01, ^**^P < 0.005, ^***^P < 0.001), analyzed by unpaired Student’s two-sided *t* test. **c** Photos of the tumors extracted from mice in different groups at the end of treatments (day 14). **d** Representative immunofluorescence images of tumor slices after hypoxia staining. The hypoxia positive areas and blood vessels were stained by HIF-1α and VEGF, respectively. H&E staining and TUNEL staining of tumor tissues at the 14th day. Nuclei were stained blue (DAPI staining), and apoptotic cells were stained red (TUNEL staining). Scale bars are 100 μm. Data were presented as mean ± s.d. (*n* = 5)

Inspired by the potency of MnO_2_@PtCo nanoflowers in vitro and the high biocompatibility in vivo, their therapeutic efficacy in vivo was first evaluated in 4T1 tumor-bearing mice via direct intratumoral injections. Twenty-eight tumor-bear mice were randomly divided into four groups, including control, MnO_2_, PtCo, and MnO_2_@PtCo. The tumor sizes of each group were measured by a caliper every other day. As presented in Fig. 8a, PtCo nanozymes exhibited certain therapeutic outcome, but failed to inhibit the growth of tumors, presumably due to the hypoxic environment of tumors^39^. Comparatively, MnO_2_@PtCo nanoflowers exhibited admirable antitumor potentials (Fig. 8b), in which the tumors were almost completely removed (Fig. 8c). These results could be ascribed to the cooperation between oxidative activity of PtCo and supply O_2_ ability of MnO_2_ components. To demonstrate the ability of MnO_2_ to ameliorate hypoxia inside tumor, HIF-1α and vascular endothelial growth factor (VEGF) staining assay were performed (Fig. 8d)^50^. MnO_2_ led to remarkable downregulation of HIF-1α, indicating tumor hypoxia was successfully relieved. However, an enhanced HIF-1α expression was observed for PtCo-treated tumor tissues, implying that PtCo worsened hypoxia in tumor. Comparatively, after MnO_2_@PtCo treatment, slight downregulation of HIF-1α was presented without affecting blood vessel densities, indicating the feasibility of our design. To further gain insight into the cytotoxic effect of nanoflowers, the tumor tissues were retrieved and processed for H&E and terminal deoxynucleotidyl transferase dUTP nick-end labeling (TUNEL) analysis. MnO_2_@PtCo nanoflowers could substantially reduce the number of tumor cells and increase the population of apoptotic cells than those control groups (Fig. 8d), indicating their capacity to inhibit tumor growth. Moreover, all the mice demonstrated negligible weight fluctuations and no obvious pathological change or other adverse effect was observed in organ tissues as compared with untreated group (Supplementary Fig. 28).

**Fig. 9:**
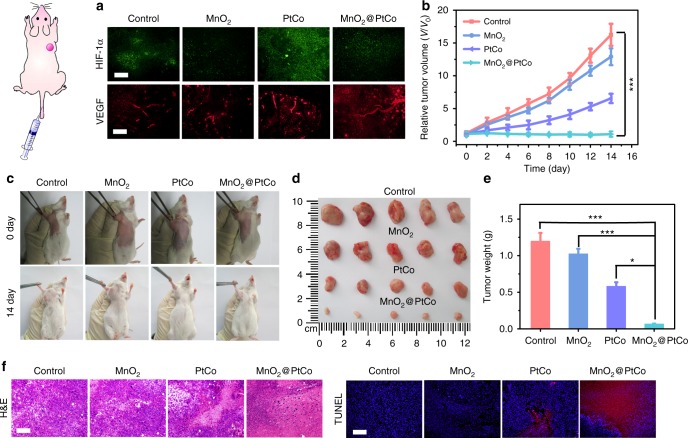
In vivo therapeutic performance of MnO_2_@PtCo nanoflowers by intravenous injection. **a** After 24 h of various nanomaterials treatments, representative immunofluorescence images of tumor slices with HIF-1α and VEGF staining. Scale bars are 100 μm. **b** Changes in tumor volumes used to evaluate the effectiveness of various treatments (i.v.) on treating tumor-bearing mice. **c** Photographs of the 4T1 tumor-bearing mice before treatment and after 14 days post injection. **d** Photos of the dissected tumors after 14 days of treatment. **e** The weight of the dissected tumors after 14 days of treatment. **f** H&E and TUNEL staining of tumor tissues at the 14th day. Scale bars are 100 μm. Asterisks indicate significant differences (^*^P < 0.01, ^**^P < 0.005, ^***^P < 0.001), analyzed by unpaired Student’s two-sided *t* test. Data were presented as mean ± s.d. (*n* = 5)

Since the different pH values between tumor tissues and normal organs as well as cancer cells are more vulnerable than normal cells to exogenous ROS, the higher and pH-dependent catalytic ability of MnO_2_@PtCo nanoflowers in modulating intracellular biochemical reactions bestow a chance to specifically suppress the growth of tumors. To prove this, we embarked on exploring the therapeutic efficacy of nanoflowers by i.v. Before that, we first studied whether MnO_2_@PtCo nanoflowers were capable of mitigating tumor hypoxia after i.v. injection. As illustrated in Fig. 9a, with the assistance of MnO_2_ nanozyme, MnO_2_@PtCo nanoflowers successfully promoted tumor oxygenation and undermine hypoxia compared to PtCo nanozymes, which was consistent with the results of intratumoral administration. On tumor suppression evaluation, the mice receiving MnO_2_@PtCo nanoflowers treatment showed remarkable inhibition effect, to a level much more effective than that of mice receiving PtCo nanozymes (Fig. 9b, c). After the therapeutic process, tumors of mice were dissected and weighted (Fig. 9d, e). These results indicated that the tumors treated with nanoflowers were almost removed, further validating their outstanding therapeutic outcome. Furthermore, H&E and TUNEL staining assays of tumors tissues further demonstrated that MnO_2_@PtCo nanoflowers caused serious apoptosis of tumor cells (Fig. 9f). Notably, negligible weight fluctuations and abnormality in the major organs of mice were observed during the whole therapeutic period (Supplementary Fig. 29), demonstrating the catalytic ability of nanoflowers was specific for tumor tissues.

## Discussion

We have developed a ROS generation platform based on self-assembled MnO_2_@PtCo nanoflowers to initiate intracellular biochemical reactions against hypoxic tumors. Compared with traditional ROS-generating systems, our platform offers a number of advantages as follows. (1) The generation of ROS was through biochemical reactions occurred in biological milieu, in which no external activation was needed, thus alleviating the problems of current ROS-mediated treatment modalities such as light penetration depth, ultrasound-induced hyperthermia, and sophisticated instruments. (2) By the cooperation between the oxidative activity of PtCo and the supply O_2_ ability of MnO_2_ components, our MnO_2_@PtCo nanoflowers were capable of relieving hypoxic condition and generating ROS efficiently in hypoxic tumors, thereby resulting in remarkable therapeutic outcome. (3) Based on cancer cells are more vulnerable to exogenous ROS than normal cells and the oxidative ability of MnO_2_@PtCo nanoflowers rely on the acidic pH (2.5–6.8), our nanoflowers could preferentially induce tumors apoptosis whereas show negligible side effect to major organs. Taken together, we expect that our approach of exploiting nanozymes assemblies as ROS source to induce cell apoptosis can open up an exciting research direction for designing and developing nanozymes to mimic intracellular enzyme for regulating cell functions such as migration, differentiation, gene expression, and so on.

## Methods

### Materials

Platinum (II) acetylacetonate (Pt(acac)_2_), Pt 48% was purchased from Alfa Aesar. Cobalt (II) acetylacetonate (Co(acac)_2_) was purchased from Adamas reagent. Poly(vinylpyrrolidone) (PVP, Mw = 8000) and 3,3,5,5-tetramethylbenzidine (TMB) were purchased from Aladdin Reagent. Benzoic acid was obtained from Macklin (Shanghai, China). 2-(*N*-morpholino)ethanesulfonic acid (MES) was purchased from Shanghai Sangon Biological Engineering Technology & Services (Shanghai, China). Potassium permanganate (KMnO_4_) and benzyl alcohol were purchased from Beijing Chemicals (Beijing, China). 2′,7′-Dichlorofuorescein diacetate (DCFH-DA), Lipid Peroxidation MDA Assay Kit, mouse monoclonal HIF-1α (clone: H1α67, catalog: AH339, 1:200), and Rabbit polyclonal anti-VEGF (catalog: AV202, 1:200) were purchased from Beyotime Biotechnology. Rabbit anti-Phospho-Histone H_2_AX (catalog: bs-3185R, 1:200) was purchased from Bioss. All chemicals were used as received from the suppliers without further purification. Ultrapure water (18.2 MΩ; Millpore Co., USA) was used throughout the experiment.

### Instruments

TEM images were recorded using a FEI TECNAI G2 20 HRTEM operating at 200 kV. XPS measurement was performed on an ESCALAB-MKII spectrometer (VG Co., United Kingdom) with Al Kα X-ray radiation as the X-ray source for excitation. XRD measurements were performed on a Bruker D8 FOCUS using Cu Kα radiation. Ultraviolet–visible (UV–vis) spectroscopy assay was recorded with a JASCO-V550 spectrometer. Fluorescence measurements were carried out using a JASCO FP-6500 spectrofluorometer with the slit width for the excitation and emission of 3 nm.

### Synthesis of PtCo nanozyme

The bimetallic PtCo nanoparticles were prepared through a one-pot strategy. The optimization composition of Pt/Co (3:1) with the highest oxidase-like activity was used in our work. Briefly, Pt(acac)_2_ (16 mg), Co(acac)_2_ (10.2 mg), benzoic acid (50 mg), and PVP (80 mg) were dispersed in 5 mL of benzyl alcohol and stirred for 15 min. Subsequently, the resultant mixture was transferred into a 10 mL Teflon-lined stainless-steel autoclave at 180 °C for 12 h. The PtCo nanoparticles products were precipitated by acetone and washed with ethanol–acetone mixture three times, and redispersed in 2 mL of deionized water as stock solution.

### Preparation of MnO_2_@PtCo nanoflowers

MnO_2_@PtCo nanoflowers were synthesized as follows. Briefly, the stock solution (20 μL, 50 μL, or 100 μL) of PtCo nanoparticles was respectively added to a 1.5-mL Eppendorf microtube (EP tube) containing 250 μL of MES buffer (0.1 M, pH 6.0). Different amounts of KMnO_4_ (10 mM, 0–200 μL, namely, 0, 50 μL, 100 μL, 150 μL, and 200 μL) was added to the EP tube. The resultant homogeneous solution was sonicated for 30 min until the solution became brown. Subsequently, MnO_2_@PtCo nanomaterials were collected by centrifugation (6000 × *g*) and washed three times with deionized water. We found that using 100 μL of PtCo nanozymes to direct the growth of MnO_2_ nanomaterials would lead to the products aggregation. The use of low concentrations of PtCo nanoparticles (20 μL) exhibited a low catalytic activity. Incredibly, when the amount of PtCo used was 50 μL, the monodisperse spherical flowers structures of MnO_2_@PtCo were prepared and exhibited the highest catalytic activity in our synthesized nanomaterials. Notably, the low concentrations of KMnO_4_ would lead to the lower field and catalytic performance. Therefore, 50 μL of PtCo and 200 μL of KMnO_4_ were used to prepare MnO_2_@PtCo nanoflowers for subsequent in vitro and in vivo studies.

### Oxidase-like activity of MnO_2_@PtCo nanoflowers

The catalytic performance of MnO_2_@PtCo nanoflowers for the oxidation of TMB was studied by monitoring the absorption spectra at 652 nm using a UV–vis spectrophotometer. In a typical test, 20 μg mL^−1^ MnO_2_@PtCo nanoflowers were suspended in 25 mM of phosphate saline buffer (PBS) (pH 5.0) and then TMB were added (final concentration 800 μM). To mimic a hypoxic condition, N_2_ gas was purged in PBS solution for 20 min to remove the dissolved O_2_ before adding MnO_2_@PtCo nanoflowers.

### Catalase (CAT)-mimic activity of MnO_2_@PtCo nanoflowers

Experiments were carried out using 20 μg mL^−1^ MnO_2_@PtCo nanoflowers in a reaction volume of 1 mL PBS buffer (pH 5.0, 6.8) with 20 mM H_2_O_2_ at 37 °C. After incubation of 10 min, the concentration of the consuming H_2_O_2_ was calculated according to the decrease of absorbance at 240 nm.

### Measurement of O_2_ production

The O_2_ concentrations in aqueous solutions were measured by a portable dissolved oxygen meter. For measurement of catalysis ability of MnO_2_ nanosheets, H_2_O_2_ was added to 30 mL of deoxygenated water and then the mixture was added in a 50 mL of three-neck flask that was closed by a rubber plug. The oxygen electrode probe was inserted through this rubber plug into the flask to measure the oxygen concentration of the solution in real time. When the O_2_ concentration of initial H_2_O_2_ solution reached equilibrium, MnO_2_ nanosheets and H_2_O_2_ were injected via a syringe into this closed system. The oxygen concentration was recorded in real time.

### Detection of superoxide radical and hydroxyl radical

The detection of O_2_^•−^ was performed by taking the interaction between hydroethidine and DNA. In the presence of O_2_^•−^, hydroethidine probe could be oxidized into ethidine which could intercalate into DNA and emit a bright red fluorescence. The determination of •OH was based on the reaction of between terephthalic acid (TA) and •OH. TA has negligible fluorescence by itself but is capable of capturing •OH and generating 2-hydroxy terephthalic acid with unique fluorescence around 435 nm.

### ESR measurements in hypoxia

For O_2_^•−^ detection, the pre-deoxidized PBS (pH 5.0, 25 mM) contained 25 mM BMPO, 20 μg mL^−1^ MnO_2_@PtCo nanoflowers, 100 μM H_2_O_2_, 50% DMSO was prepared. After incubation of 5 min, ESR spectra were recorded. For •OH detection, the pre-deoxidized PBS (pH 5.0, 25 mM) contained 25 mM BMPO, 20 μg mL^−1^ MnO_2_@PtCo nanoflowers, 100 μM H_2_O_2_, 0.25 U mL^−1^ SOD was prepared. After incubation of 5 min, ESR spectra were recorded. The following instrument settings were used for collecting ESR spectra: 1 G field modulation, 100 G scan range, and 20 mW microwave power.

### Cell culture in normoxic and hypoxic environments

Murine breast cancer 4T1 cell (ATCC-CRL-2539) and mouse embryonic fibroblast NIH 3T3 cell (ATCC-CRL-1658) were purchased from China Center for Type Culture Collection. Both 4T1 and NIH 3T3 cells were cultured with regular growth media contained with high glucose DMEM. The cell growth media was supplemented with 10% FBS, 100 U mL^−1^ penicillin, and 100 mg mL^−1^ streptomycin and cultured at 37 °C in a 5% CO_2_ for mimicking the normoxic environment. The media was changed every 2 days and the cells were digested by trypsin and re-suspended in fresh medium before confluence. In order to mimic the hypoxic tumor microenvironment, 4T1 and NIH 3T3 cells were cultured in a DMEM medium in an atmosphere of 1% O_2_ and 5% CO_2_ at 37 °C. Notably, throughout the studies, all cells were tested negative for mycoplasma contamination.

### Cell internalization at 4 °C

Briefly, 4T1 cells were randomly plated in six-well plates and allowed to adhere for 24 h. After that, MnO_2_@PtCo nanoflowers (100 μg mL^−1^) and PtCo nanoparticles (100 μg mL^−1^) were added into the six-well plates, respectively. Subsequently, the six-well plates were placed in 4 °C refrigerator. After incubation at different times, the 4T1 cells in six-well plates were washed with PBS three times and then harvested and treated with aqua regia for 8 h under heat treatment (80 °C) for dissolution of intracellular nanoparticles. The obtained liquid was subjected to ICP-MS analysis.

### Determination of ROS generation in vitro

For ROS detection, 4T1 cells were randomly seeded in six-well plates in DMEM for 24 h before further manipulation. Then cells were then incubated with MnO_2_ (100 μg mL^−1^), PtCo nanozymes (100 μg mL^−1^), and MnO_2_@PtCo nanoflowers (100 μg mL^−1^) for 12 h under normoxic and hypoxic conditions, respectively. The treated cells were washed with PBS twice and incubated with 10 μM of DCFH-DA for 30 min. After the unloaded probe was removed with PBS, the fluorescence intensity of cells was monitored by flow cytometry and fluorescence microscopy.

### In vitro cytotoxicity assay

MTT assay was used to determine the cytotoxicity of MnO_2_@PtCo nanoflowers under normoxia and hypoxia. Briefly, 4T1 cells were randomly seeded into 96-well plates at a density of 5000 cells per well (100 μL), under 100% humidity and were cultured at 37 °C with 5% CO_2_ for 24 h. Different concentrations of MnO_2_@PtCo nanoflowers were added to the wells. The cells were subsequently incubated for 48 h in the CO_2_ incubator under normoxic and hypoxic conditions, respectively. Then, 10 μL of MTT solution (5 mg mL^−1^) was added to each well to a final volume of 100 μL. After that, the plate was placed in the CO_2_ incubator for additional 4 h. The media was removed and DMSO (100 μL) was added into each well. Absorbance values were determined with Bio-Rad model-680 microplate reader at 490 nm (corrected for background absorbance at 630 nm). The cell viability was estimated according to the following equation: Cell Viability (%) = (OD_Treated_/OD_Control_) × 100%. Where OD_Control_ was obtained in the absence of nanoparticles, and OD_Treated_ was in the presence of nanoparticles.

### Annexin V/PI assay

4T1 cells were randomly plated in six-well plates and allowed to adhere for 24 h in a hypoxic environment (1% O_2_). Then the cells were incubated with MnO_2_ (100 μg mL^−1^), PtCo (100 μg mL^−1^), and MnO_2_@PtCo nanoflowers (100 μg mL^−1^) for 24 h. After that, the cells were stained with 5 μL annexin V-FITC at room temperature for 15 min and followed by 10 μL PI for 5 min in the dark. The fluorescence intensity of cells was viewed by flow cytometry in green channel for annexin V-FITC and red channel for PI, respectively.

### MCTS experiments

The three-dimensional MCTS were constructed as follows. Briefly, 5 × 10^4^ 4T1 cells per well were seeded into a 1.5% (w/v) agarose pre-coated 96-well plate and incubated at 37 °C in humidified atmosphere with 5% CO_2_ for 10 days to form MCTS. The culture medium was replaced with fresh DMEM containing 10% FBS every 2 days.

To demonstrate our system capable of alleviating hypoxic microenvironment, the MCTS were treated with PBS, MnO_2_ (100 μg mL^−1^), PtCo (100 μg mL^−1^), and MnO_2_@PtCo (100 μg mL^−1^) for 24 h, respectively. Afterwards, the immunofluorescence staining of HIF-1α in treated MCTS was performed. Firstly, MCTS was fixed in 4% paraformaldehyde for 1 h, and then treated with 0.5% Triton X-100 for 30 min, followed by bovine serum albumin (BSA) was used to prevent other nonspecific protein interactions. Subsequently, the cells were incubated with HIF-1α mouse monoclonal antibody (1:200) overnight at 4 °C, and then the corresponding FITC-labeled sheep anti-mouse secondary antibody (1:500) was added for 2 h at 37 °C. Hoechst was used to stain the cell nuclei. Finally, the resultant MCTS was observed by confocal laser scanning microscope. The obtained images were collected at the center of each MCTS.

We also studied the ROS generation ability and toxicity of MnO_2_@PtCo nanoflowers in MCTS. The MCTS were treated with PBS, MnO_2_ (100 μg mL^−1^), PtCo (100 μg mL^−1^), and MnO_2_@PtCo (100 μg mL^−1^) for 24 h, respectively. Afterwards, the MCTS was fixed in 4% paraformaldehyde for 1 h, and then incubated with DCFH-DA for 1 h to determine the ROS generation efficacy. Furthermore, after fixing the MCTS treated with different samples in 4% paraformaldehyde for 1 h, they were stained with LIVE/DEAD Kit with 1 h to investigate their cytotoxicity.

### Determination of lipid peroxidation

Lipid peroxidation was evaluated by measuring methane dicarboxylic aldehyde (MDA) production based on TBA reactivity. In a typical experiment, 4T1 cells were seeded in six-well plates and cultured in a hypoxic environment for 24 h and then the adherent cells were randomly incubated with MnO_2_ (100 μg mL^−1^), PtCo (100 μg mL^−1^), and MnO_2_@PtCo (100 μg mL^−1^) for 24 h, respectively. After that, the adherent cells were harvested and treated according to the lipid peroxidation MDA Assay Kit.

### DNA double-strand breaks under hypoxia

To test DNA double-strand breaks, 4T1 cells were randomly seeded in a 24-well plate and incubated in a hypoxic environment for 24 h. Then different treatments including MnO_2_ (100 μg mL^−1^), PtCo (100 μg mL^−1^), and MnO_2_@PtCo (100 μg mL^−1^) were added into the 24-well plate. After treatment for 24 h, the immunofluorescence staining of γ-H_2_AX was conducted. Firstly, the cells were fixed with 4% paraformaldehyde for 30 min and subsequently 0.5% Triton X-100 was used to penetrate the cells for 5 min, followed by BSA was used to prevent other nonspecific protein interactions. Finally, the cells were incubated with γ-H_2_AX antibody (1:200) overnight at 4 °C and then the corresponding FITC-labeled sheep anti-mouse secondary antibody (1:500) was added for 1 h at 37 °C. Excess antibody was removed by rinsing coverslips in PBS. Hoechst was used to stain the cell nuclei.

### Analysis of lysosomal disruption

The ROS-induced lysosomal damage was tested using acridine organe (AO) as an indicator. AO emitted red fluorescence in acidic organelle such lysosomes while emitted green fluorescence in cytoplasm and nuclei. Typically, 4T1 cells were randomly seeded in a 24-well plate and incubated in a hypoxic environment for 24 h. Subsequently, different groups including MnO_2_ (100 μg mL^−1^), PtCo (100 μg mL^−1^), and MnO_2_@PtCo (100 μg mL^−1^) were added into the wells for incubation of 24 h. Then, the cells were stained with AO (5 μM) for 15 min and were washed twice with 1× PBS. Then cells were images immediately by fluorescence microscopy under excitation 488 nm.

### Analysis of MMP

4T1 cells were randomly planted in six-well plates and allowed to adhere for 24 h in a hypoxic environment and the adherent cells were incubated with MnO_2_ (100 μg mL^−1^), PtCo (100 μg mL^−1^), and MnO_2_@PtCo (100 μg mL^−1^) for 24 h, respectively. Then, the cells were harvested and treated with JC-1 (5 μg mL^−1^) at 37 °C for 20 min. The fluorescence intensity of the cells was measured by flow cytometry and fluorescence microscopy, respectively. Cells were viewed on fluorescence microscopy in red channel for J-aggregates and green channel for JC-1 monomer, respectively.

### In vivo biocompatibility of MnO_2_@PtCo nanoflowers

Eight-week-old female Balb/c mice were purchased form Laboratory Animal Center of Jilin University (Changchun, China) and all animal care and handing procedures were in accordance with the guidelines approved by the ethics committee of Jilin University. All study protocols involving animals were approved by the Jilin University Animal Care and Use Committee. Thirty healthy female Balb/c mice were randomly divided into three groups, including saline (control), MnO_2_ nanoflowers (5 mg kg^−1^, 7 days post injection), and MnO_2_ nanoflowers (5 mg kg^−1^, 28 days post injection). Aliquots (50 μL) of saline and nanoflowers were intravenously administrated into mice of the corresponding group. The mice body weights were measured every 3 days to evaluate the in vivo biosafety. At the indicated time, the mice were euthanized and their blood samples were collected to perform complete blood panel analysis and serum biochemistry assay at the Jilin ADICON Clinical Laboratories, Inc. The major organs containing heart, liver, spleen, lung, and kidney were harvested, fixed in 10% paraformaldehyde, processed into paraffin, sectioned at ~4 μm, and stained with H&E.

### In vivo biodistirbution and pharmacokinetic of MnO_2_@PtCo

In the quantitative biodistribution analysis, 4T1 tumor-bearing mice were intravenously injected with MnO_2_@PtCo nanoflowers (50 μL, 5 mg kg^−1^) and randomly divided into three groups (*n* = 3). At 4 h, 8 h, and 24 h post administration, the mice were sacrificed, and the tumor tissues and main organs containing heart, liver, spleen, lung, and kidney were excised and weighted, and then digested in aqua regia under heat treatment (80 °C) for 3 days to analyze the content of Pt^2+^ in the samples using ICP-MS.

For in vivo pharmacokinetic evaluation, female Balb/c mice (*n* = 3) were i.v. injected with MnO_2_@PtCo nanoflowers (50 μL, 5 mg kg^−1^). At 5, 10, 30 min, 1, 2, 4, 8, 12 h, and 24 h post injection, 10 μL of blood were collected by puncturing the tail vein and dispersed into 990 μL aqua regia to dissolve the remained MnO_2_@PtCo nanoflowers. The concentrations of Pt ions were measured by ICP-MS. The in vivo circulating half-life of MnO_2_@PtCo in blood stream (*τ*_1/2_) is calculated by a double-compartment pharmacokinetic model. The eliminating rate curve was conducted by plotting ln(*C*_pt_) against time and fitted according to the two-compartment models. The eliminating rates were reflected by the slopes of the curve.

### Tumor model and MnO_2_@PtCo nanoflowers treatment in vivo

To develop a tumor model, 4T1 cells (1 × 10^6^) suspended in PBS were subcutaneously injected into the left flank mammary gland of each female Balb/c mouse. When the tumor volume reached ≈ 100 mm^3^, mice were divided randomly into four groups consisting of seven mice in each group. Then, mice received 50 μL of the intratumoral and i.v. of PBS, MnO_2_ (5 mg kg^−1^), PtCo (5 mg kg^−1^), and MnO_2_@PtCo nanoflowers (5 mg kg^−1^), respectively. Subsequently, tumor growth was recorded by measuring the tumor’s perpendicular diameter using a caliper estimated by employing the following equation: volume = (tumor length) × (tumor width)^2^/2. To ensure the accuracy of experiments, three individuals participated in the experiments. One was responsible for preparing nanozymes, selecting mice and handing them to the second researcher who performed the injections. The last researcher collected and analyzed results. So, investigators performing tumor measurements were blinded to treatment groups.

### Immunofluorescence stainings of HIF-1α and VEGF

4T1 tumor-bearing mice were divided into four groups randomly, and subjected to the following treatments: group 1: PBS (50 μL) only; group 2: MnO_2_ (50 μL, 5 mg kg^−1^); group 3: PtCo (50 μL, 5 mg kg^−1^); group 4: MnO_2_@PtCo nanoflowers (50 μL, 5 mg kg^−1^). After 24 h post injection, the tumors of mice with a similar tumor volume in these four groups were collected and fixed in 10% paraformaldehyde, embedded in paraffin, sectioned into ~4 μm and treated with 0.5% Triton X-100 for 10 min, followed by incubation of BSA for 30 min. Then, the slices were incubated with HIF-1α antibody (1:200) and VEGF antibody (1:200) overnight at 4 °C, respectively. Subsequently, the corresponding secondary antibody (1:500) was added for 1 h at 37 °C. Excess antibody was removed by rinsing coverslips in PBS.

### H&E and TUNEL staining

For histology, major organs and tumors were harvested from mice at 48 h after injection. The collected tumors and organs were fixed in 10 % paraformaldehyde, embedded in paraffin, sectioned into ~4 μm, and stained with H&E. In addition, for determining tumor cells apoptosis, tumor slices were stained with TUNEL according to One Step TUNEL Apoptosis Assay Kit (purchased from Beyotime Biotechnology). The histology was performed in college of Basic Medical Sciences of Jilin University. The samples were examined by an Olympus BX-51 microscope.

### Statistical analysis

All data were expressed in this article as mean result ± standard deviation (s.d.). All figures shown in this article were obtained from five independent experiments with similar results unless specific mention. The statistical analysis was performed by using Origin 8.0 software. Statistical evaluation was performed using unparied Student’s two-sided *t* test analysis. Asterisks indicate significant differences (^*^P < 0.01, ^**^P < 0.005, ^***^P < 0.001). Notably, no samples and animals were excluded from the analysis.

### Data availability

All data are available from the authors on reasonable request.

## Electronic supplementary material

Supplementary Information
