# Supplementary material for: Biomimetic nanoflowers by self-assembly of nanozymes to induce intracellular oxidative damage against hypoxic tumors
Source: Nat Commun. 2018 Aug 20;9:3334. doi: 10.1038/s41467-018-05798-x (PMC6102211; doi:10.1038/s41467-018-05798-x)
Supplement: Supplementary file 1 — Supplementary Information [file 41467_2018_5798_MOESM1_ESM.pdf]

Supplementary Information

Biomimetic nanoflowers by self-assembly of nanozymes to induce intracellular oxidative damage against hypoxic tumors

Wang et al.

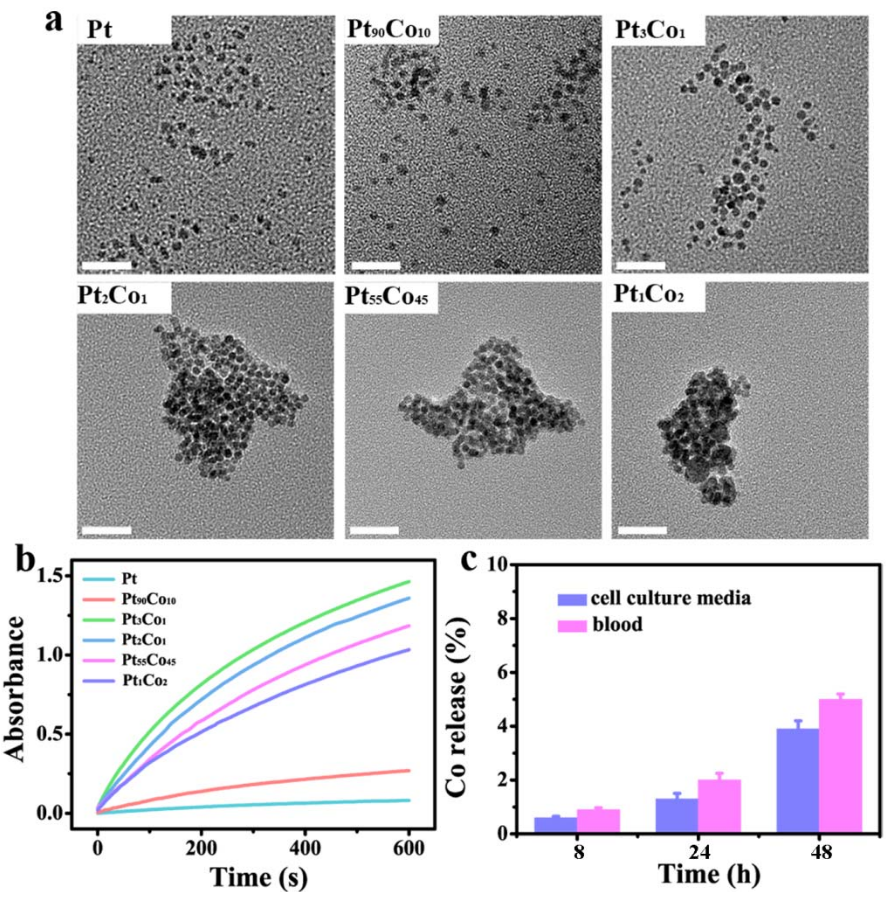

Supplementary Figure 1. The effect of Pt/Co ratio on the enzyme-mimic activity of nanoparticles. a) Representative TEM images of as-prepared PtCo nanoparticles with different molar ratios. Scale bars are 100 nm. b) The enzymatic activity of PtCo nanoparticles with different molar ratios. c) Percentage of Co^{2+} ions released from PtCo (3 : 1) nanoparticles after 8, 24 and 48 h of incubation in cell culture medium and blood measured by ICP-MS. Data were presented as mean ± s.d. (n = 5).

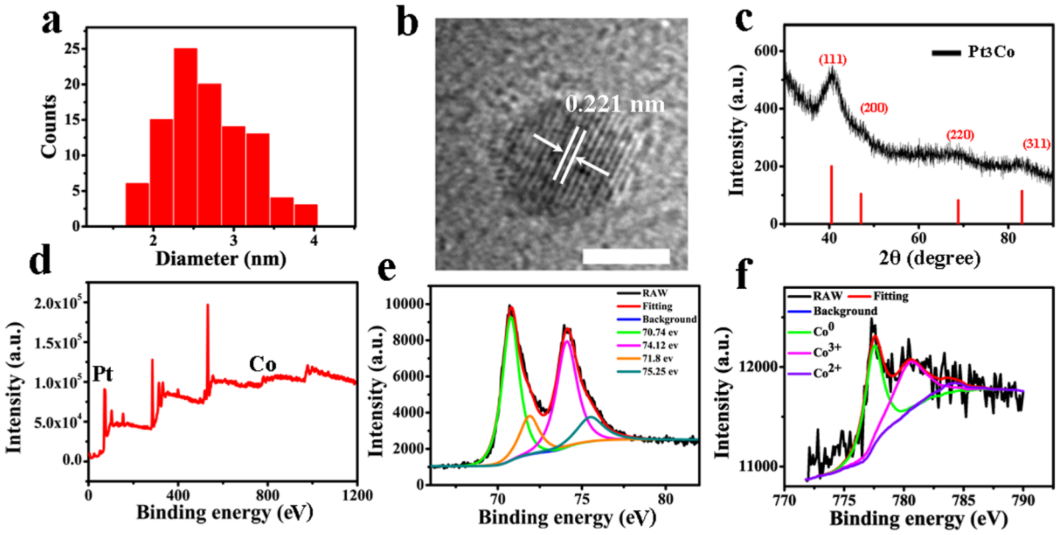

Supplementary Figure 2. Characterizations of PtCo nanozymes. a) Size distribution histogram of PtCo nanoparticles. The diameter was statistically calculated based on 200 nanoparticles. b) HRTEM images of PtCo nanoparticles. Scale bars are 3 nm. c) X-ray diffraction pattern of Pt_3Co (abbreviate as PtCo in this manuscript) and the standard pattern of PtCo nanoparticles. d) XPS spectra of the well-synthesized PtCo nanoparticles. e), f) Pt 4*f* and Co 2*p* core level spectra of PtCo nanoparticles, respectively. The Pt 4*f* spectrum was deconvoluted into two pairs of doublets because of the spin-orbit splitting of 4*f* states. The most intense doublet with binding energies of 74.1 and 70.7 eV represented the zerovalent metallic state, and the rest doublet at binding energies of 71.8 and 75.3 eV indicated the existence of + 2 oxidation state. The Co 2*p* spectra were fit with up to three peaks: Co metal, Co^{2+}, and Co^{3+}. The XPS results indicated that both Pt and Co were mainly in the form of metallic state, demonstrating the formation of PtCo bimetallic nanoparticles.

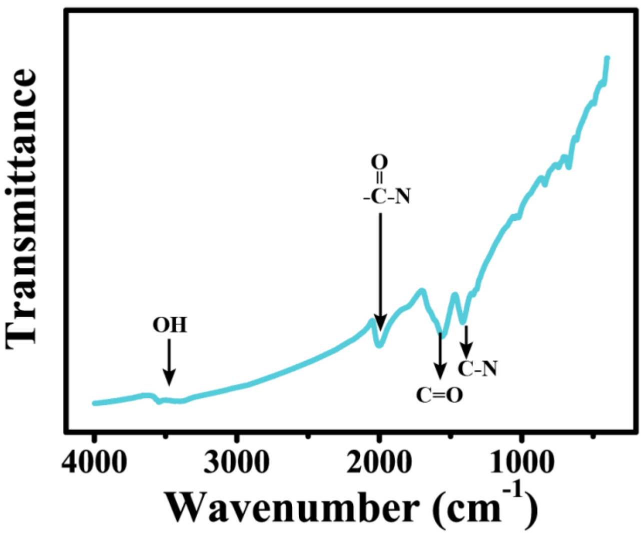

Supplementary Figure 3. FTIR spectra of PtCo nanoparticles with abundant –COOH, -C-N, -OH functional groups.

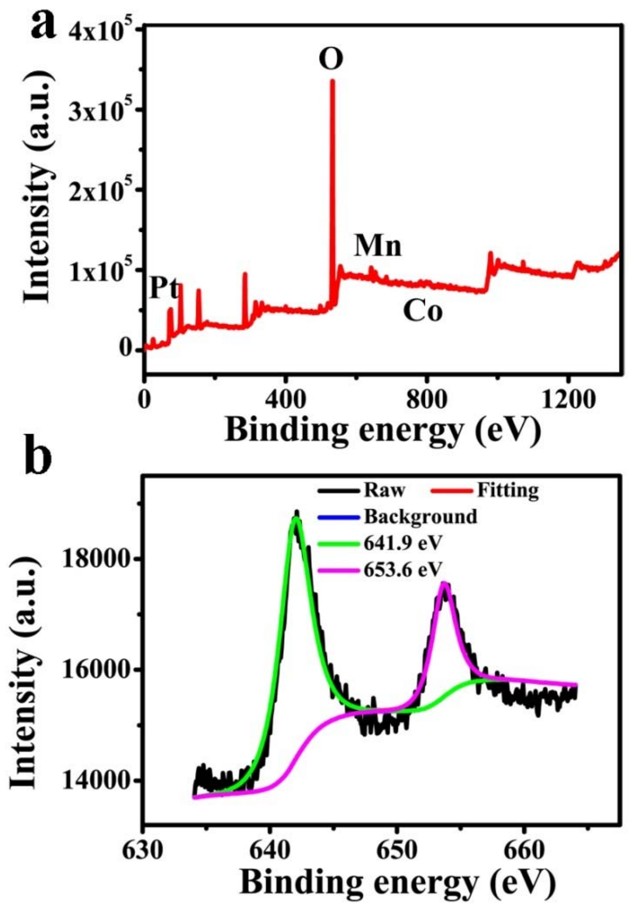

Supplementary Figure 4. XPS analysis of nanoflowers. a) XPS spectra of the well-synthesized MnO_2@PtCo nanoflowers. b) Mn 2*p* core level spectra of MnO_2@PtCo nanoflowers.

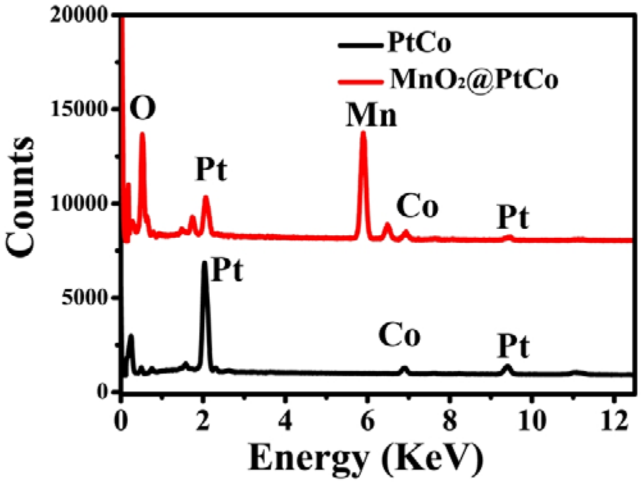

Supplementary Figure 5. EDX spectra of PtCo and MnO_2@PtCo nanoflowers, respectively.

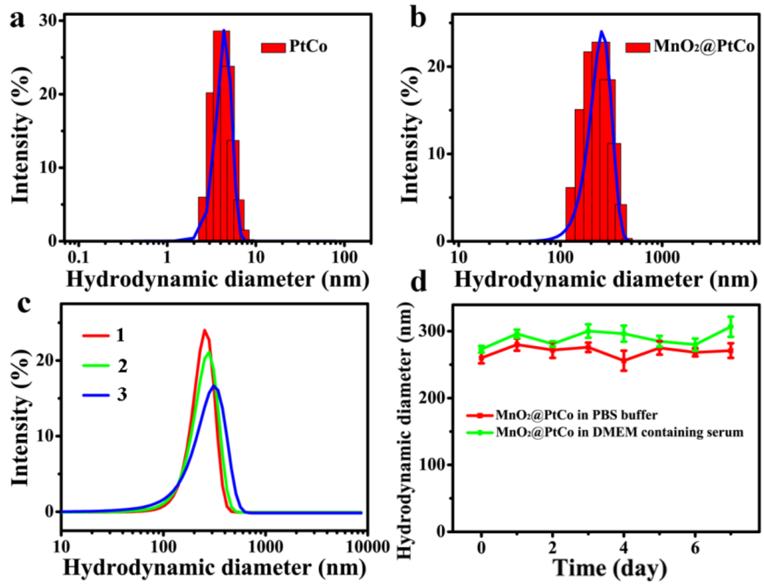

Supplementary Figure 6. **Hydrodynamic diameter distribution of the prepared nanoparticles.** a), b) Hydrodynamic diameter of PtCo nanoparticles and MnO_2@PtCo nanoflowers measured in water, respectively. c) The size distribution of MnO_2@PtCo nanoflowers measured in water (1), PBS (2) and DMEM containing 10% fetal bovine serum (FBS) (3). d) The stability of MnO_2@PtCo nanoflowers in PBS buffer and DMEM containing 10% FBS, respectively. Data were presented as mean ± s.d. (n = 5).

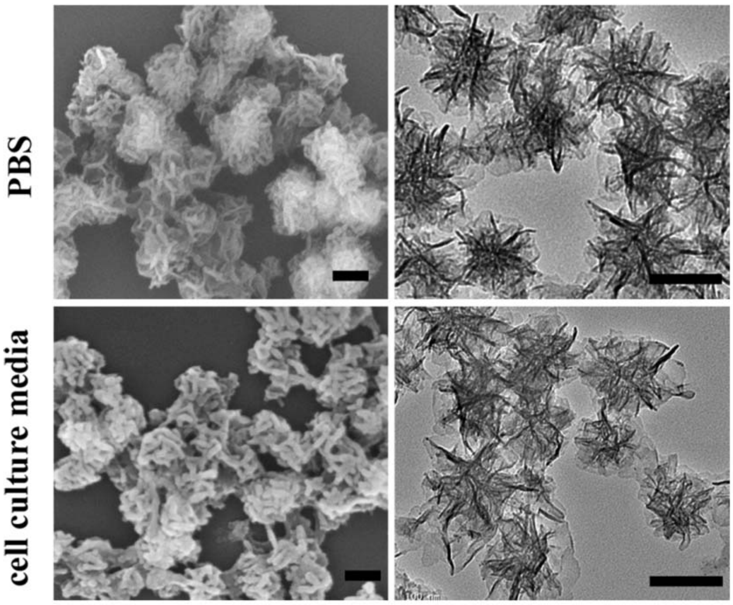

Supplementary Figure 7. The morphology stability of MnO_2@PtCo nanoflowers. SEM and TEM images of MnO_2@PtCo nanoflowers after 7 days of incubation in PBS and DMEM containing 10% FBS (cell culture media), respectively. Scale bars are 100 nm.

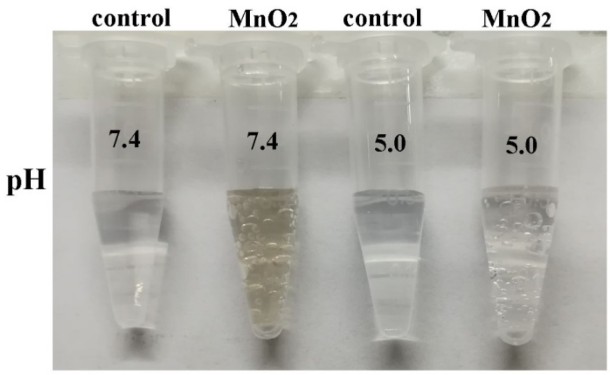

Supplementary Figure 8. The O_2 generation ability of MnO_2 in different pH vaules. Digital photos of generation gas bubbles after 10 min incubation of MnO_2 nanosheets with H_2O_2 in different pH values. In addition to catalyzing H_2O_2 into O_2 in neutral condition, MnO_2 nanosheets were able to produce O_2 from H_2O_2 via a redox reaction in acidic condition. Such appealing properties made MnO_2 components suitable for overcoming hypoxia in tumors.

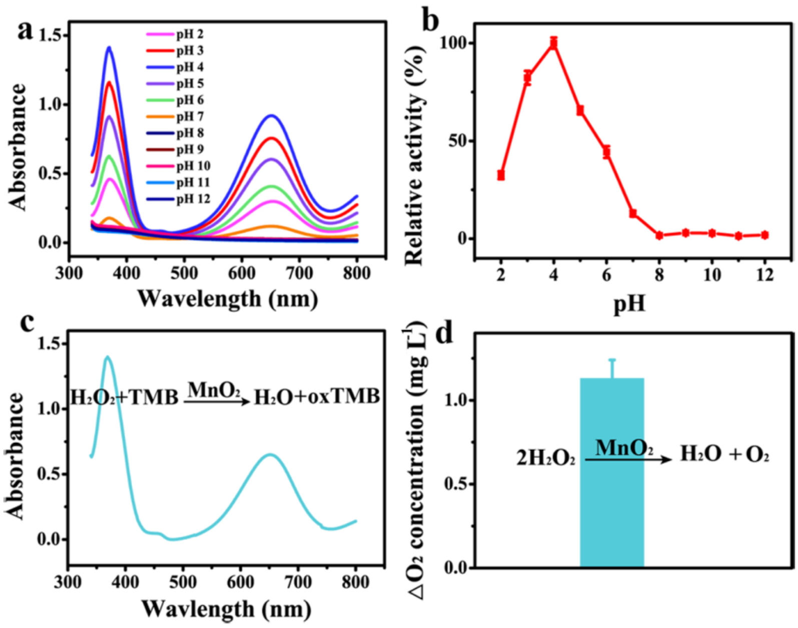

Supplementary Figure 9. The peroxisase-like activity of MnO_2. a) The peroxidase-like activity of MnO_2 component with pH range 2-12 in the presence of H_2O_2 (100 μM) after 5 min incubation. b) The peroxidase activity of MnO_2 component is pH-dependent. c) The peroxidase activity of MnO_2 under 100 μM of H_2O_2 (pH 6.8) after 30 min incubation. d) The changes of dissolved O_2 of H_2O_2 (100 μM) catalyzed by MnO_2 in pH 6.8 after 30 min incubation. Data were presented as mean ± s.d. ($n = 5$).

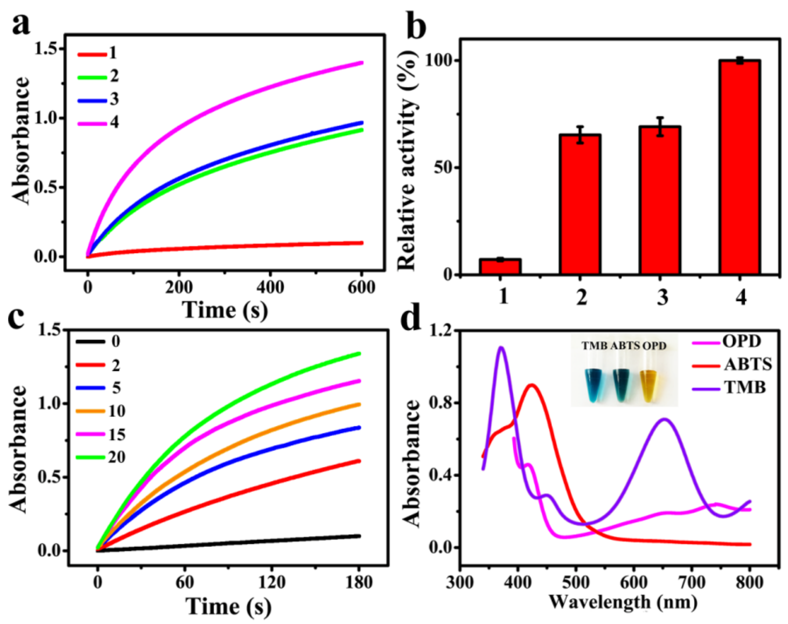

Supplementary Figure 10. The oxidase-like activity of MnO_2@PtCo nanoflowers. a) Comparison of catalytic activities of MnO_2@PtCo nanoflowers and the physical mixtures of MnO_2 and PtCo: 1) MnO_2, 2) PtCo, 3) MnO_2 + PtCo, 4) MnO_2@PtCo nanoflowers. b) Relative activity of MnO_2@PtCo nanoflowers and physical mixtures of MnO_2 and PtCo: 1) MnO_2, 2) PtCo, 3) MnO_2 + PtCo, 4) MnO_2@PtCo nanoflowers. Data were presented as mean ± s.d. (n = 5). c) Time-dependent absorbance changes of TMB with different concentrations of MnO_2@PtCo nanoflowers. d) MnO_2@PtCo nanoflowers could catalyze the substrates, TMB, 2,2′-azino-bis(3-ethylbenzthiazoline-6-sulfonic acid) (ABTS) and o-phenylenediamine (OPD) to produce blue, green and yellow color respectively.

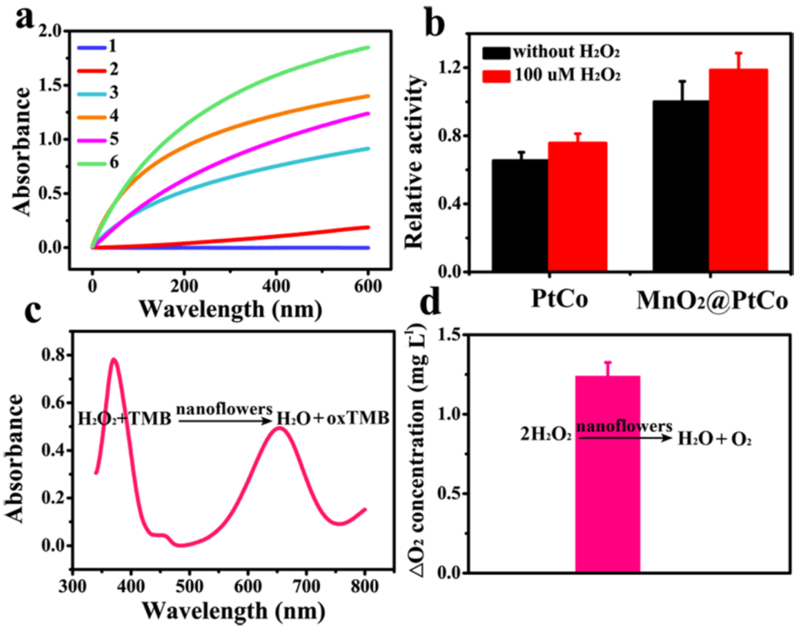

Supplementary Figure 11. The peroxisase-like activity of MnO_2@PtCo nanoflowers. a) Comparison of catalytic activities of PtCo nanoparticles with MnO_2@PtCo nanoflowers in the presence of H_2O_2 (100 μM) or not: 1) control, 2) H_2O_2, 3) PtCo, 4) MnO_2@PtCo, 5) PtCo + H_2O_2; 6) MnO_2@PtCo + H_2O_2. b) Relative catalytic activities of PtCo nanoparticles and MnO_2@PtCo nanoflowers with/without H_2O_2. c) The peroxidase activity of MnO_2@PtCo nanoflowers under 100 μM of H_2O_2 (pH 6.8) after 30 min incubation. d) The changes of dissolved O_2 of H_2O_2 (100 μM) catalyzed by MnO_2@PtCo nanoflowers in pH 6.8 after 30 min incubation. Data were presented as mean ± s.d. (*n* = 5). According to the Beer-Lambert Law (A = εbc), the concentration of oxidized TMB was calculated to be 12.6 μM, indicating 12.6 % of H_2O_2 was involved in the peroxidase reaction of MnO_2@PtCo nanoflowers. Comparatively, the concentration of O_2 was determined to increase by 38.7 μM, suggesting 77.4 % of H_2O_2 was involved in the catalase reaction of MnO_2@PtCo nanoflowers.

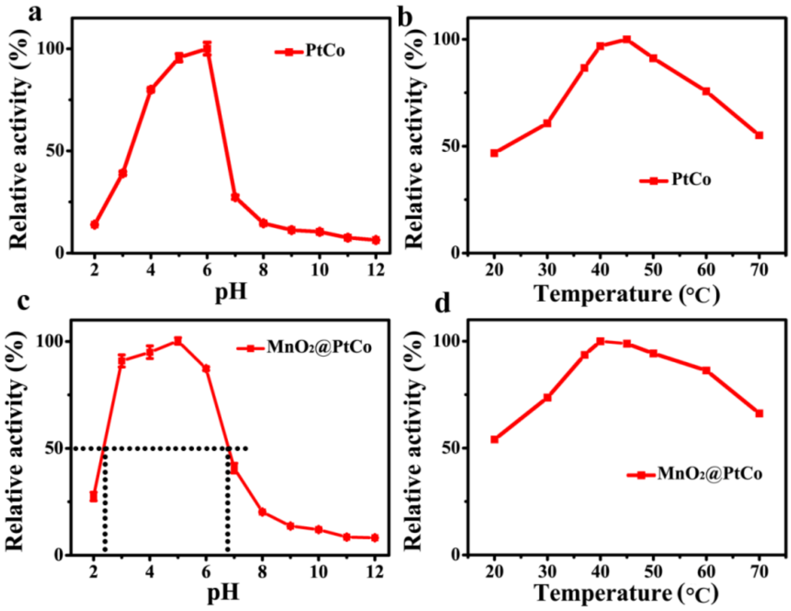

Supplementary Figure 12. The influence of pH and temperature on oxidase-like activity of nanozymes. The influence of pH (a) and temperature (b) on catalytic ability of PtCo nanoparticles. The catalytic activity of MnO_2@PtCo nanoflowers is dependent on pH (c) and temperature (d). Data were presented as mean ± s.d. (n = 5).

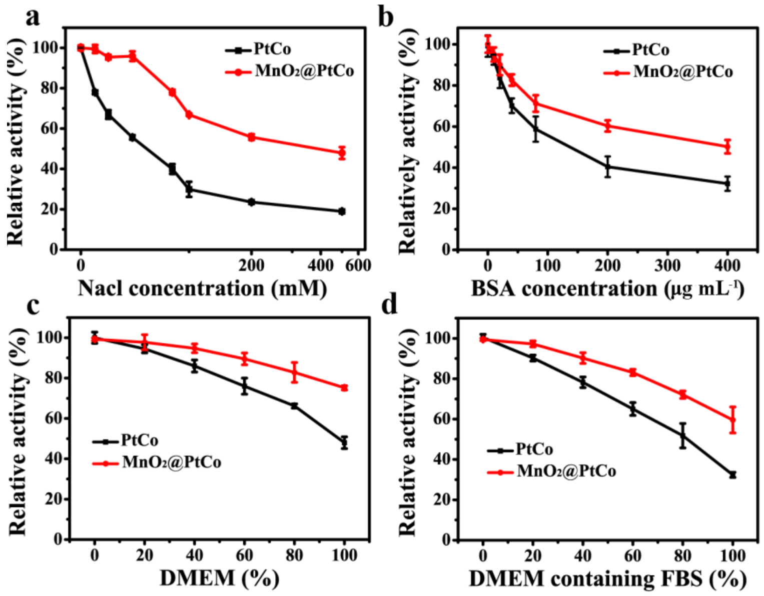

Supplementary Figure 13. The stability of nanozymes in physiological conditions. a) The influence of NaCl on the enzymatic activity of MnO_2@PtCo nanoflowers. b) Relative catalytic ability of MnO_2@PtCo nanoflowers in the presence of bovine serum protein (BSA) at various concentrations. c), d) Relative enzymatic activity of nanoflowers in DMEM and DMEM with FBS at indicated conditions, respectively. Data were presented as mean ± s.d. (n = 5).

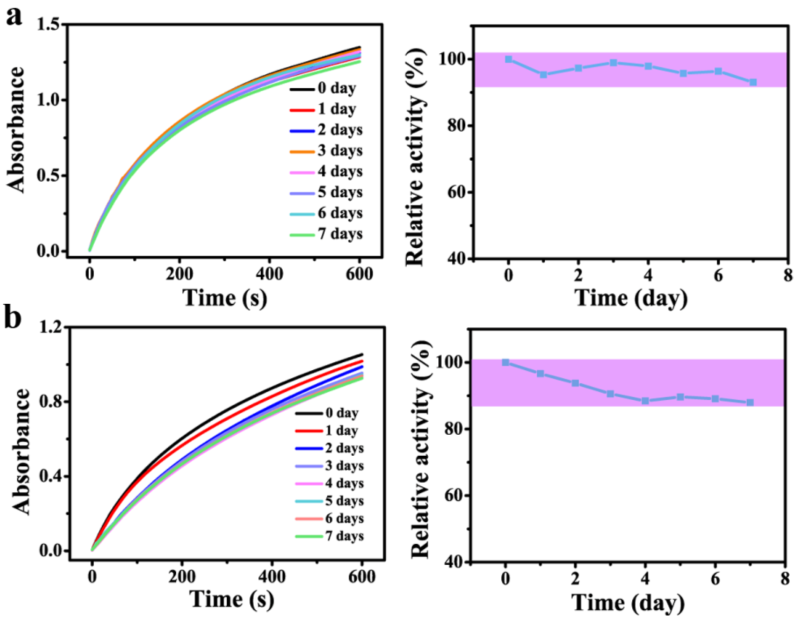

Supplementary Figure 14. The catalytic stability of MnO_2@PtCo nanoflowers. The catalytic ability of MnO_2@PtCo nanoflowers after incubating PBS buffer (a) and DMEM containing 10% FBS (b) with different time.

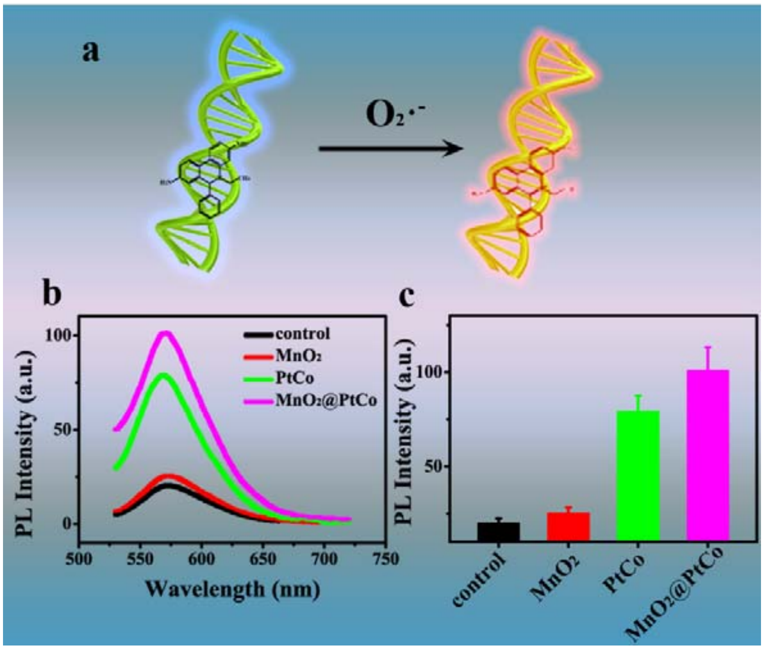

Supplementary Figure 15. The $O_2^{\bullet-}$ generation ability of nanozymes. a) Schematic illustrated of detection of $O_2^{\bullet-}$ by hydroethidine in the presence of DNA. b). Fluorescence spectra of PBS buffer included hydroethidine and DNA (control); hydroethidine and DNA incubated with different treatments for 12 h. c) The corresponding fluorescence intensity of hydroethidine and DNA at 573 nm after various treatments. Data were presented as mean ± s.d. (n = 5). Compared with control and MnO_2 treatment, both PtCo and MnO_2@PtCo induced remarkable fluorescence enhancement at 573 nm, indicating the production of $O_2^{\bullet-}$.

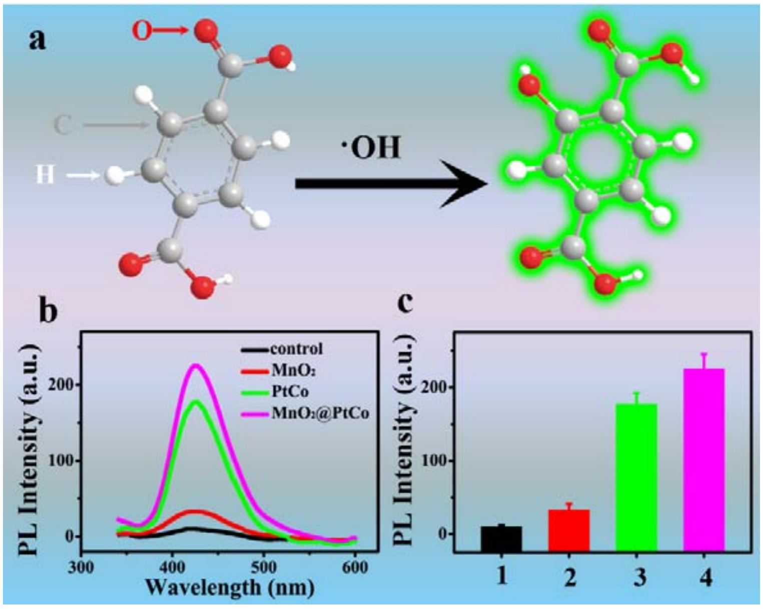

Supplementary Figure 16. The ·OH generation ability of nanozymes. a) Schematic representation of detection of ·OH by terephthalic acid (TA). b) Fluorescence spectra of PBS buffer included TA (control); TA and MnO_2 nanosheets; TA and PtCo nanoparticles; TA and MnO_2@PtCo nanoflowers after 12 h incubation. c) The corresponding fluorescence intensity of TA at 435 nm in different groups: control (1); MnO_2 (2); PtCo nanoparticles (3); MnO_2@PtCo nanoflowers (4). Data were presented as mean ± s.d. (n = 5). Compared with control and MnO_2 treatment, both PtCo and MnO_2@PtCo induced remarkable fluorescence enhancement at 435 nm, indicating the generation of ·OH.

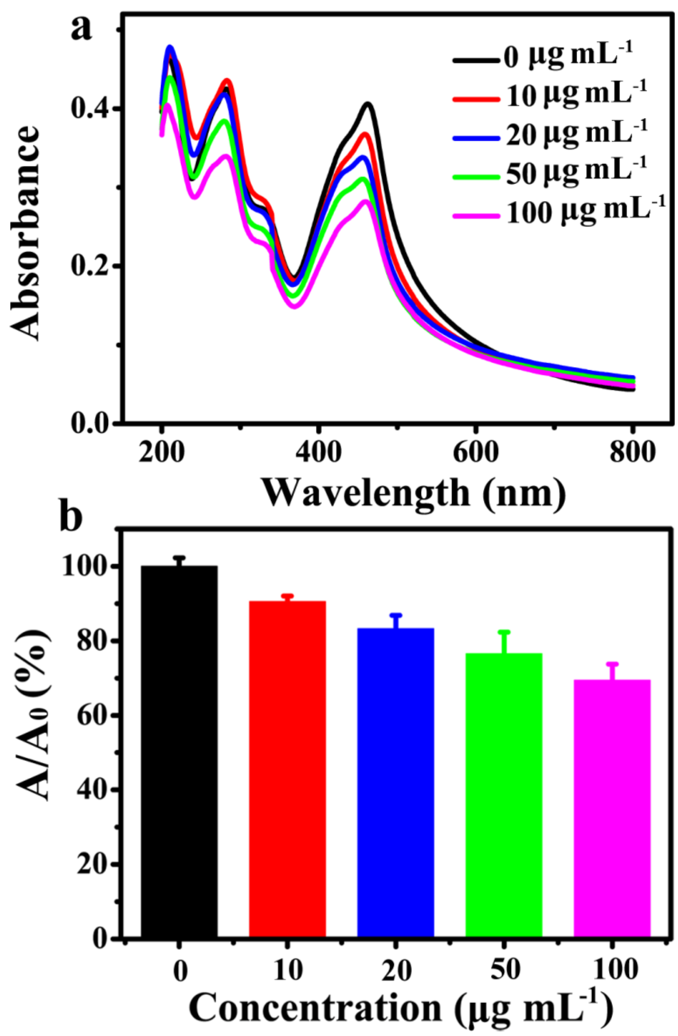

Supplementary Figure 17. The $^1O_2$ generation ability of nanozymes. a) UV-vis spectra of DPBF incubated with different concentrations of MnO_2@PtCo nanoflowers. b) The remained DPBF percent after treating with different concentrations of MnO_2@PtCo nanoflowers. A_0 is the original absorbance of DPBF probe at 460 nm. Data were presented as mean ± s.d. (n = 5).

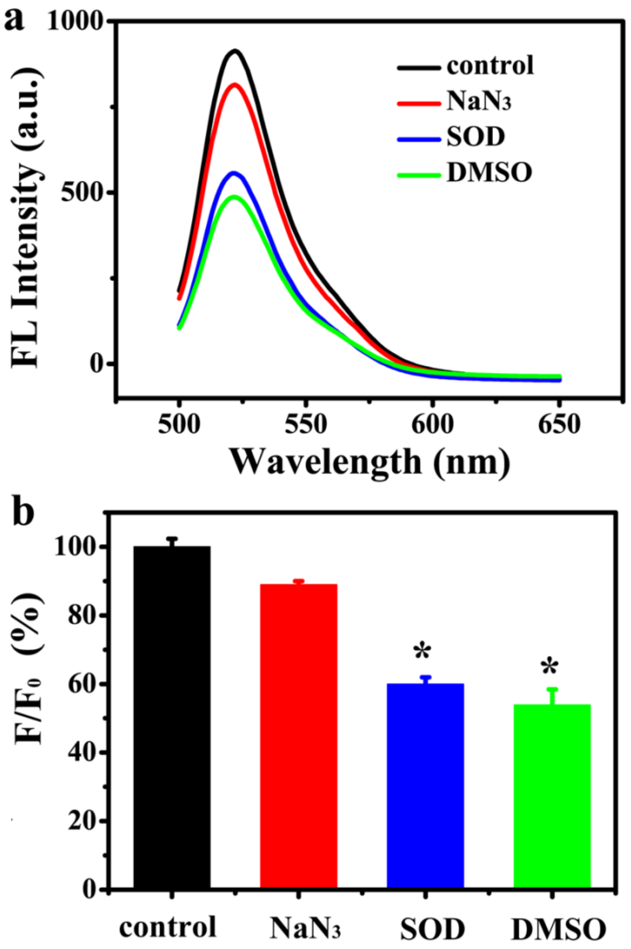

Supplementary Figure 18. Identifying main kinds of generated ROS by MnO_2@PtCo nanoflowers. a) Fluorescence spectra of DCFH-DA incubated with MnO_2@PtCo nanoflowers in the presence of different inhibitors. b) Fluorescence intensity percent of DCFH-DA treated with MnO_2@PtCo nanoflowers in the presence of various scavengers. F_0 is the fluorescence intensity of DCFH-DA treated with nanoflowers at 525 nm. Asterisks indicate significantly differences ($^{*}P < 0.01$, $^{**}P < 0.005$, $^{***}P < 0.001$), analyzed by unpaired Student's two-sided *t*-test. Data were presented as mean ± s.d. ($n = 5$).

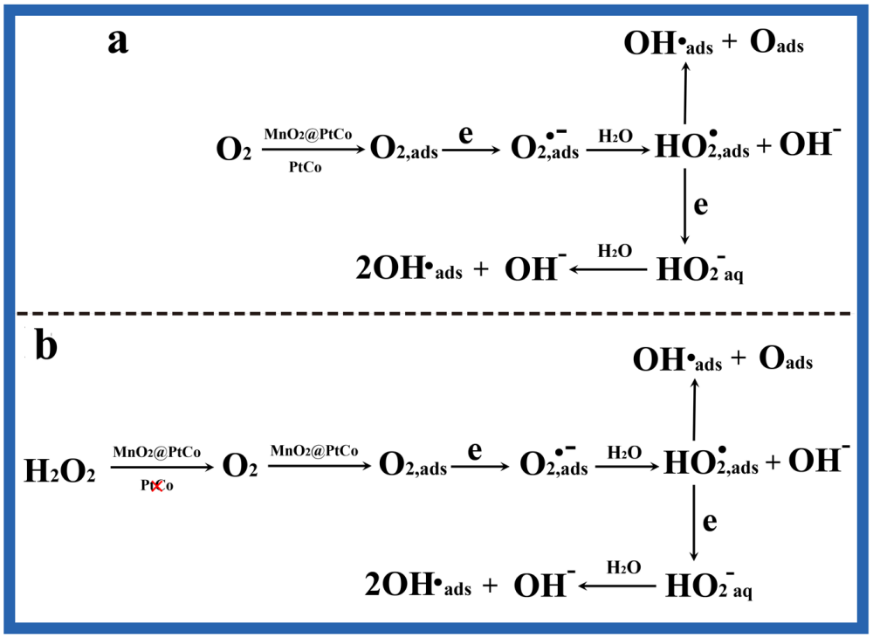

Supplementary Figure 19. The possible oxidase-like mechanism of nanozymes. a) In normoxic conditions, oxygen adsorption on the nanoflowers surface is firstly involved in the initial electron transfer step with the reduction of O_2 to the superoxide anion; secondly, the superoxide anion is not stable in water and forms an $HO_2^{\bullet}$ radical upon protonation. Thirdly, the $HO_2^{\bullet}$ radical is unstable and dissociates into $OH\cdot_{ads}$ and O_{ads}, or it can also be reduced to HO_2^- and further reduced to hydroxyl radical. Hence, both the hydroxyl radical and the superoxide anion are active oxygen species in the present catalytic system. b) The possible mechanism for generation of superoxide anion and hydroxyl radical by MnO_2@PtCo nanoflowers and PtCo in a hypoxic tumor microenvironment. Unlike in normoxia, MnO_2@PtCo nanoflowers firstly catalyze H_2O_2 into O_2, and then catalyze O_2 to generate superoxide anion and hydroxyl radical under hypoxic conditions. As PtCo nanozyme cannot catalyze H_2O_2 into O_2, it exhibits ineffective oxidase-like activity in hypoxic conditions.

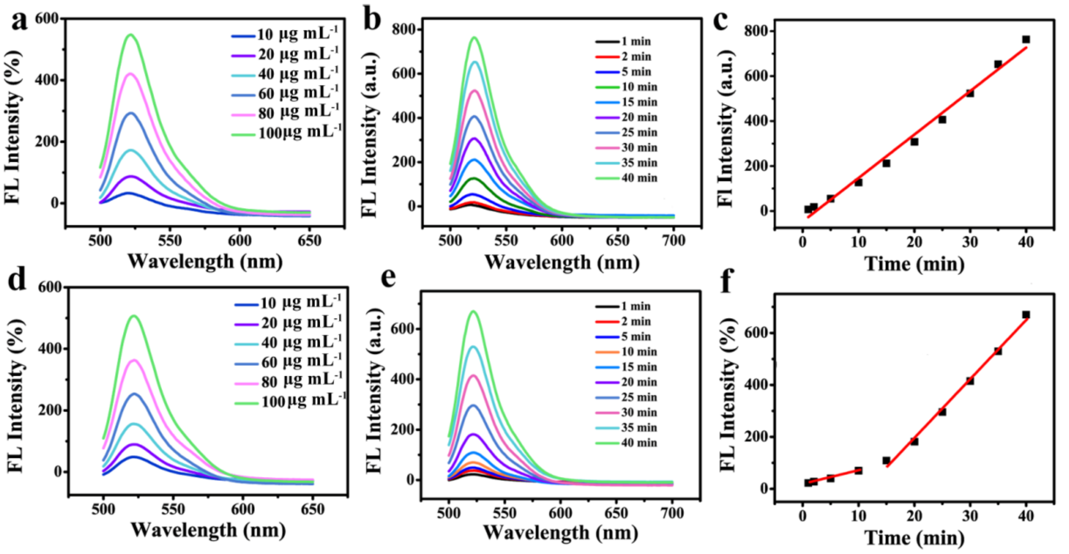

Supplementary Figure 20. The ROS generation ability of MnO_2@PtCo nanoflowers. Fluorescence spectra of DCFH incubated with different levels of MnO_2@PtCo nanoflowers in normoxia (a) and hypoxia (d). Fluorescence spectra of DCFH incubated with nanoflowers at different time points in normoxia (b) and hypoxia (e). The linear plots of fluorescence intensity measured at 525 nm as a function of time in normoxia (c) and hypoxia (f).

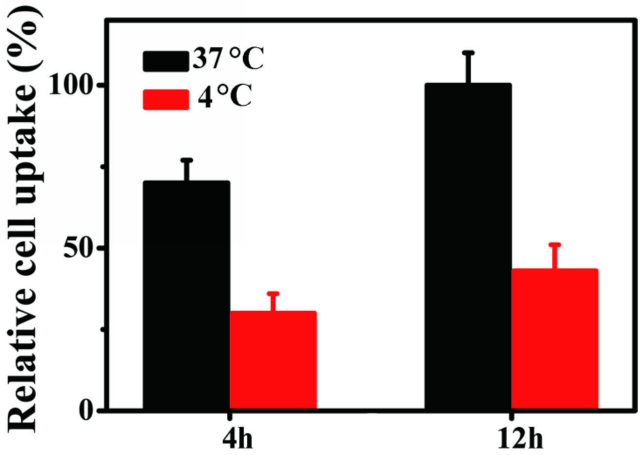

Supplementary Figure 21. The cellular uptake of 4T1 cells treated with MnO_2@PtCo nanoflowers in different temperatures. Data were presented as mean ± s.d. ($n = 5$).

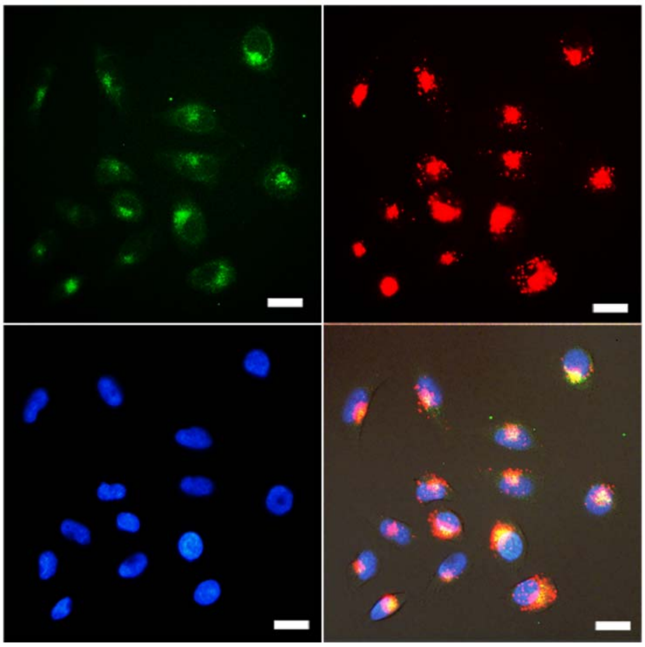

Supplementary Figure 22. Co-localization images of MnO_2@PtCo nanoflowers in 4T1 cells. The FITC-labeled sDNA was absorbed on the surface of MnO_2@PtCo nanoflowers to investigate cell internalization of nanoflowers. After 4h of incubation, the lysosomes and cell nuclei were stained with LysoTracker Red and DAPI, respectively. Scale bars are 20 μm.

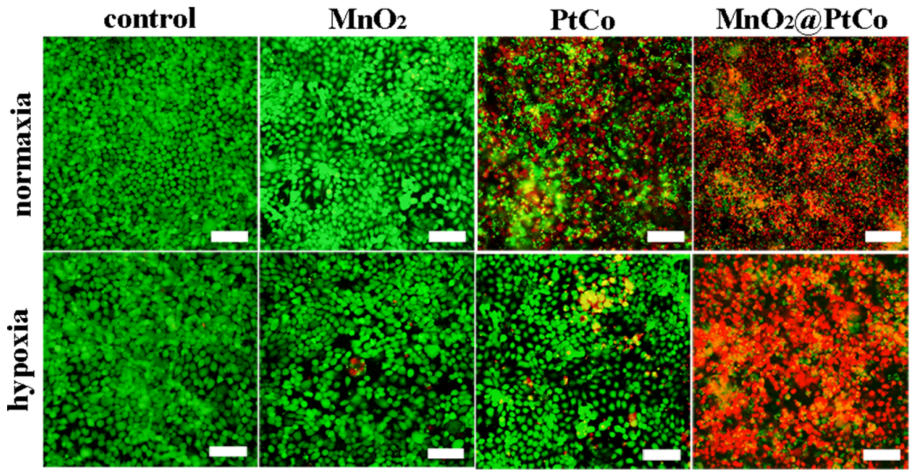

Supplementary Figure 23. LIVE/DEAD staining of 4T1 cells incubated with different nanoparticles in both normoxic and hypoxic conditions. Scale bars are 100 μm.

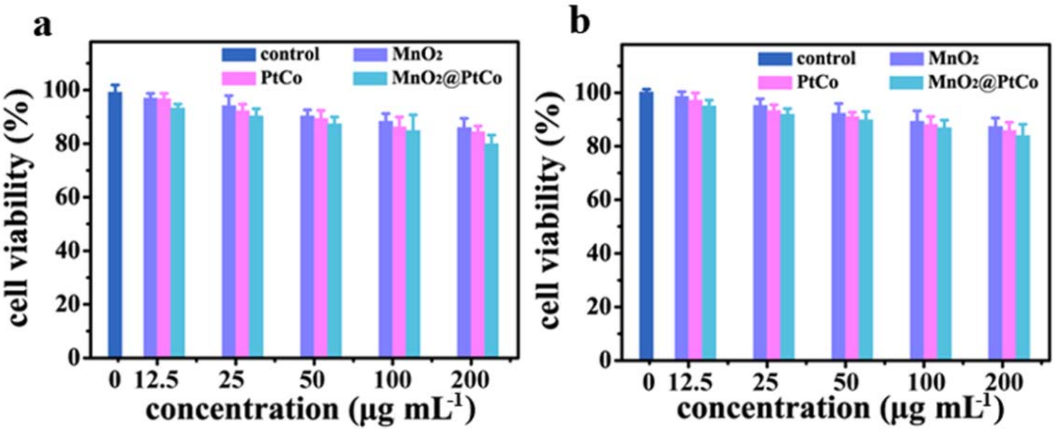

Supplementary Figure 24. *In vitro* biocompatibility of nanozymes. Cell viability of NIH 3T3 cells treated with different concentrations of nanoparticles for 48 h in both normoxic (a) and hypoxic (b) conditions. Data were presented as mean ± s.d. (n = 5).

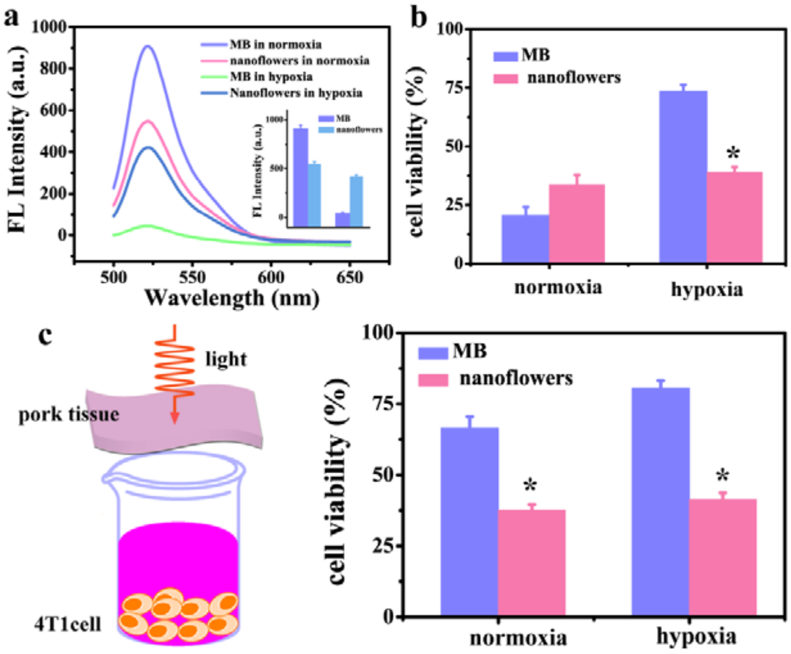

Supplementary Figure 25. Comparison of therapeutic efficacy of MnO_2@PtCo nanoflowers with photosensitizer. a) The fluorescence spectra of DCFH-DA treated with methylene blue (MB) under light irradiation of 30 min at 638 nm with a power density of 500 mW cm^{-2} and MnO_2@PtCo nanoflowers in both normoxia and hypoxia. The inset is the fluorescence intensity of DCFH-DA at 525 nm after different treatments. b) Cytotoxicity of MB under light and MnO_2@PtCo nanoflowers in both normoxia and hypoxia. c) Cell viability induced by MB under light and MnO_2@PtCo nanoflowers in both normoxia and hypoxia through 4 mm of pork tissue. Asterisks in Supplementary Figure 25 indicate significantly differences ($^{*}P < 0.01$, $^{**}P < 0.005$, $^{***}P < 0.001$), analyzed by unpaired Student's two-sided *t*-test. Data were presented as mean ± s.d. ($n = 5$).

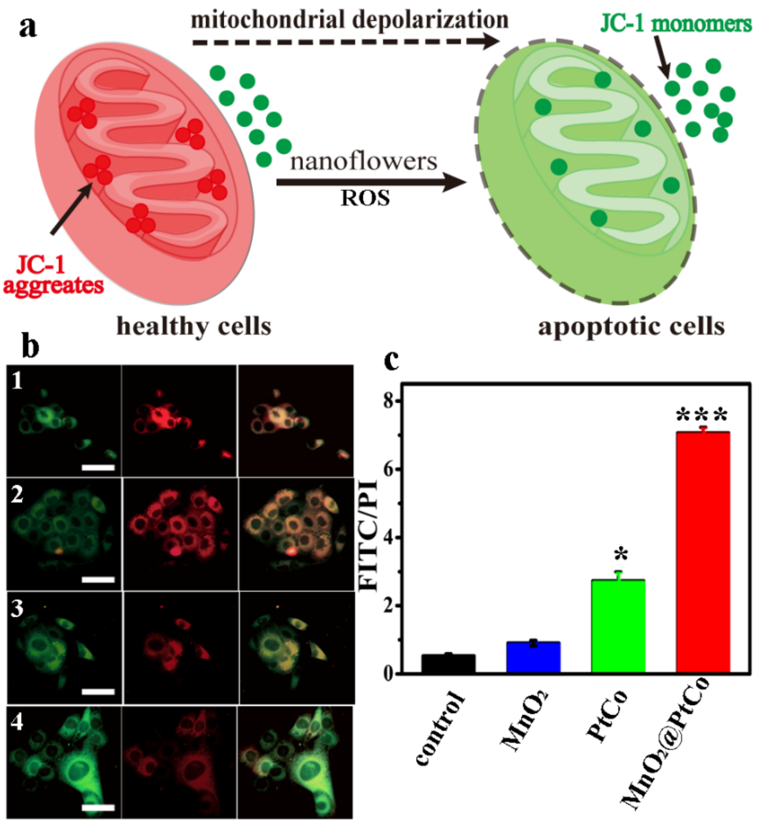

Supplementary Figure 26. The influence of nanozymes on mitochondrial depolarization of 4T1 cells. a) Schematic diagram depicting the presence of JC-1 aggregates (red; healthy cells) and JC-1 monomers (green; apoptotic cells) as a result of MnO_2@PtCo nanoflowers treatment. b) Mitochondrial membrane potential of 4T1 cells was measured with fluorescence microscopy after different treatments: control (1); MnO_2 (2); PtCo (3); MnO_2@PtCo (4). Scale bars are 50 μm. c) The ratio of fluorescent intensity of JC-1 aggregates (FITC) to JC-1 mononers (PI) insides 4T1 cells incubated with different treatments, as determined by flow cytometry. Asterisks indicate significantly differences ($^{*}P < 0.01$, $^{**}P < 0.005$, $^{***}P < 0.001$), analyzed by unpaired Student's two-sided t-test. Data were presented as mean ± s.d. ($n = 5$).

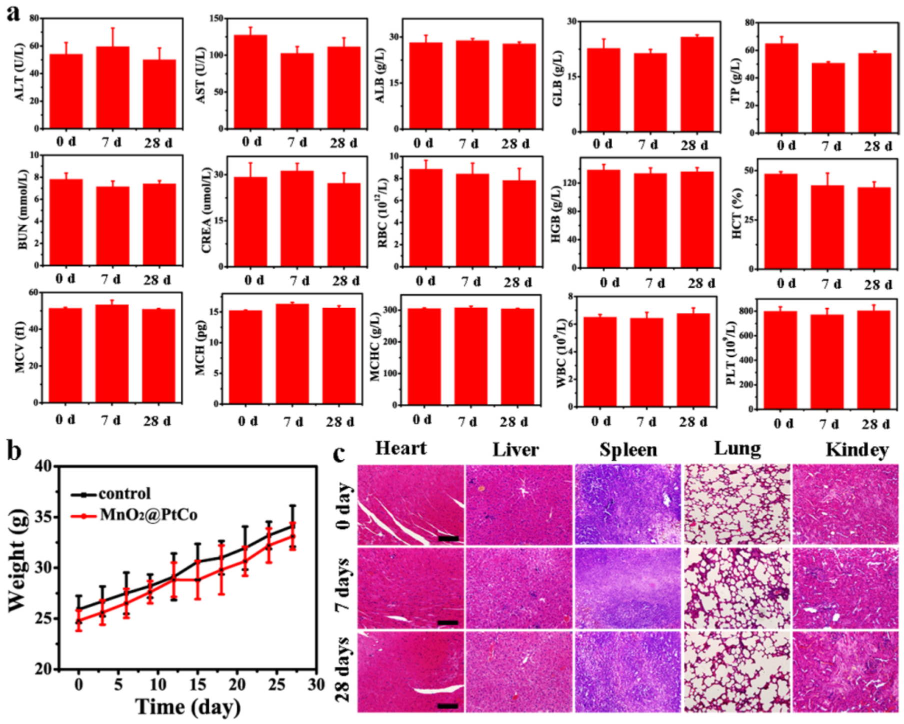

Supplementary Figure 27. ***In vivo* toxicity.** a) Blood biochemical and haematological analysis of the healthy mice intravenously injected with MnO_2@PtCo nanoflowers (5 mg Kg^{-1}) at 7 and 28 days post-injection. b) Body weights of mice treated with the nanoflowers, record every three days. c) H&E images obtained from the major organs of the nanoflowers-treated mice at 7 and 28 days post-injection. Scale bars are 100 µm. Data were presented as mean ± s.d. (n = 5).

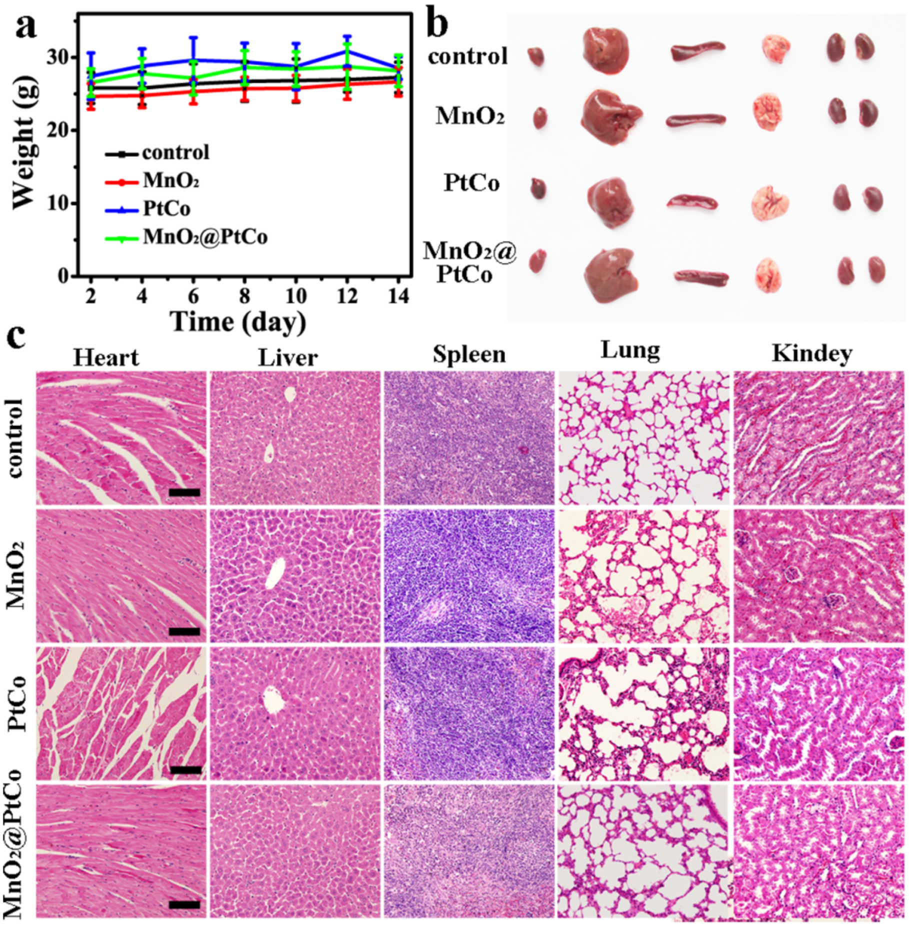

Supplementary Figure 28. *In vivo* biosafety evaluation of MnO_2@PtCo nanoflowers by intratumoral injection. a) Time-dependent body-weight curves of mice after different treatments. Data were presented as mean ± s.d. (n = 5). b) Representative digital image of major organs after 14 days of treatment with various materials. c) Histopathological examinations via H&E staining of major organs after 14 days of treatment with various materials. Scale bars are 100 μm.

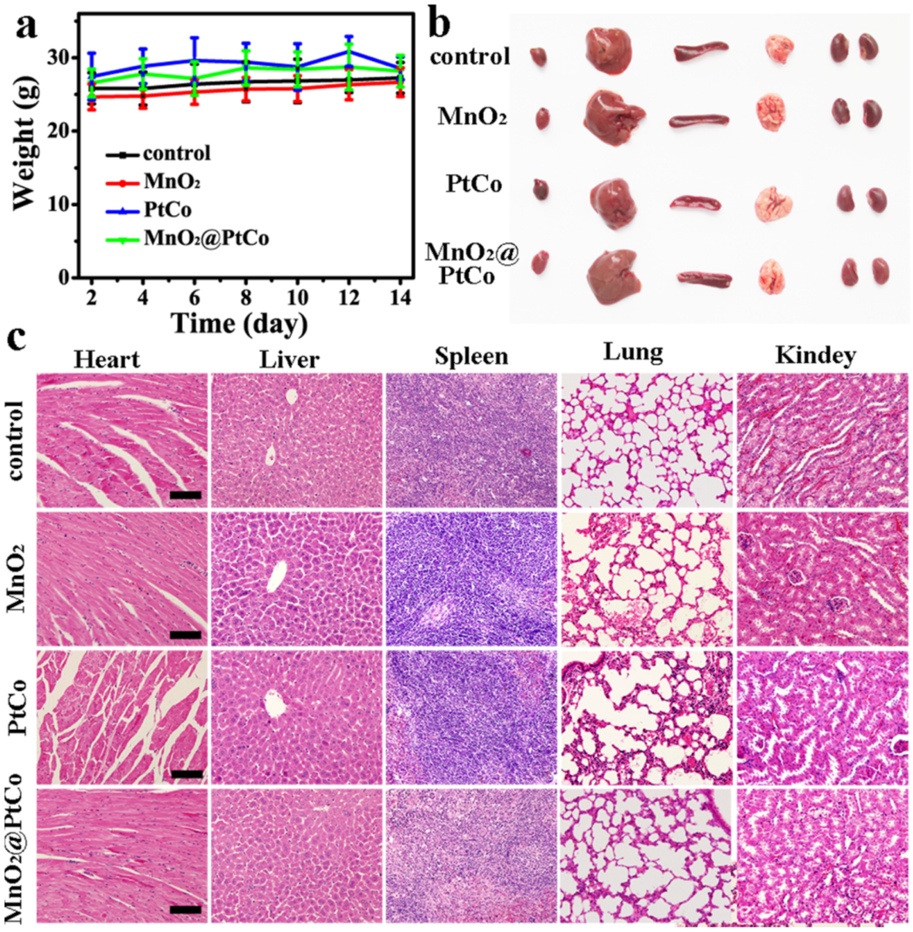

Supplementary Figure 29. The biocompatibility of MnO_2@PtCo nanoflowers by intravenous infection. a) Time-dependent body-weight curves of mice after different treatments. Data were presented as mean ± s.d. (n = 5). b) Representative digital image of major organs after 14 days of treatment with various materials. c) Histopathological examinations via H&E staining of major organs after 14 days of treatment with various materials. Scale bars are 100 μm.
